# Three new compounds with nitric oxide inhibitory activity from *Tirpitzia sinensis*, an ethnomedicinal plant from Southwest China

**DOI:** 10.1186/s13065-019-0568-9

**Published:** 2019-04-01

**Authors:** Ronghui Gu, Yuehu Wang, Shibiao Wu, Yeling Wang, Ping Li, Li Xu, Yue Zhou, Ze’e Chen, Edward J. Kennelly, Chunlin Long

**Affiliations:** 1 College of Life and Environmental Sciences, Minzu University of China, 27 Zhongguancun South Ave., Haidian, Beijing, 100081 People’s Republic of China; 2 Kunming Institute of Botany, Chinese Academy of Sciences, 132 Lanhei Road, Heilongtan, Kunming, 650201 People’s Republic of China; 3 Department of Biological Sciences, Lehman College, City University of New York, 250 Bedford Park Boulevard West, Bronx, New York, 10468 USA; 4 Ph.D. Programs in Biology, The Graduate Center, City University of New York, 365 Fifth Ave., New York, 10016 USA; 5 Key Laboratory of Ethnomedicine, Minzu University of China, Ministry of Education, 27 Zhongguancun South Ave., Haidian, Beijing, 100081 People’s Republic of China

**Keywords:** *Tirpitzia sinensis*, Linaceae, Lignans, Inflammatory, NO inhibition, Pharmacological targets, Pharmacophore, In silico

## Abstract

The medicinal plant *Tirpitzia sinensis* has been used by the Zhuang ethnic people in mountainous areas of Southwest China to stop bleeding, invigorate blood circulation, and treat inflammation and wounds. In order to further explore its traditional medicinal uses, the phytochemical constituents of this species were examined. Three new compounds, the lignan tirpitzin (**1**), the flavonoid tirpitzoside (**2**), and the furan-glycoside tirpitziol (**3**), along with five known compounds were isolated from the aerial part of *T. sinensis* for the first time. The structures of these compounds were elucidated by 1D and 2D NMR, LC/MS, IR spectrometric methods and compared with published data. The results of an in silico pharmacophore-based analysis showed potential targets of the new compounds, including ERBB2, IRAK4, LCK, JAK2, MAPK14, and MMP-12. These targets suggested that **1**–**3** may be involved with wound-healing and/or inflammation, leading to an in vitro assay of nitric oxide (NO) inhibition assays with lipopolysaccharide-induced BV-2 cells. All three new compounds displayed moderate NO inhibitory activity with the IC_50_ values of 14.97 ± 0.87, 26.63 ± 1.32, and 17.09 ± 2.3 μM, respectively.

**Electronic supplementary material:**

The online version of this article (10.1186/s13065-019-0568-9) contains supplementary material, which is available to authorized users.

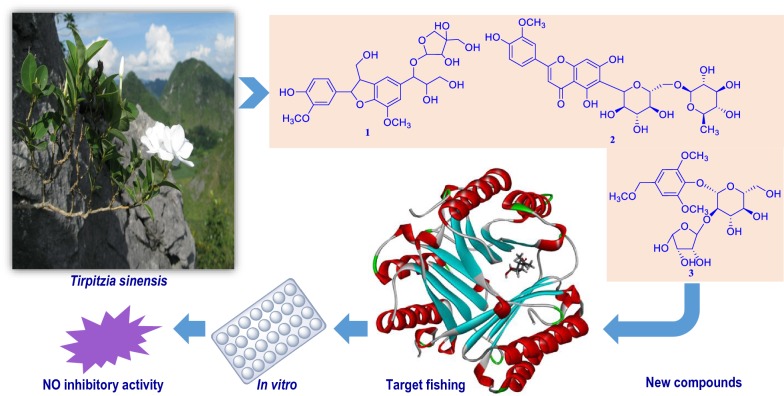

## Introduction

*Tirpitzia sinensis* (Hemsl.) Hallier f., known in China as “qing li chai”, is a shrub or small tree in the Linaceae family. It is distributed mainly on the geologically distinct highly exposed karst limestone hills and low mountains in Guangxi, Guizhou, and Yunnan provinces of Southwest China and northern Vietnam. The branches and leaves of *T. sinensis* have been used traditionally to treat swelling, alleviate pain, and set fractures [1]. Based on our ethnobotanical survey of traditional herbs market of Jingxi County in Guangxi during 2012 and 2013, we found that the Zhuang people used *T. sinensis* as a medicinal plant to stop bleeding, invigorate blood circulation, and treat inflammation and traumatic injury. Flavonoids [2], cyanogenic glucosides [3], and lignans [4, 5] have been isolated from the closely related and well-studied genus *Linum* (Linaceae). However, no phytochemical constituents nor biological activity has been reported for *T. sinensis*. Therefore, as part of our continuing ethnobotanical study of Chinese folk medicinal plants, we investigated the chemical constituents of *T. sinensis,* and explored their potential biological activity.

Screening the bioactivity of *T. sinensis* constituents is a first step to evaluate scientifically the rationale of its traditional medicinal uses. Biological screening can be complex since the assay system to be used is not always obvious. The assays chosen can be time- and cost-intensive and the success rate may not be high [6]. In recent years, pharmacophore-based parallel screening approaches have been used successfully to predict pharmacological targets of small molecules [7, 8]. Pharmacophore models are a series of three-dimensional arrangements of essential chemical features, which represent the interaction between a ligand/compound and its binding site of the receptor/pharmacological target [9].

Inflammation is the response to tissue injury, swelling and host protection [10]. NO has been reported to be involved in some inflammatory disorders, including chronic hepatitis, rheumatoid arthritis, and pulmonary fibrosis, and inhibition of NO release was considered as one of the promising ways to treat these diseases [11]. Lipopolysaccharide (LPS)-induced NO production is a common in vitro model for discovery of anti-inflammatory agents [12], which have been applied to estimate anti-inflammatory activity of new compounds [13–15]. BV-2, a microglia cell lines, applied to extensive research related to inflammation, especially in neuroinflammation.

**Fig. 1:**
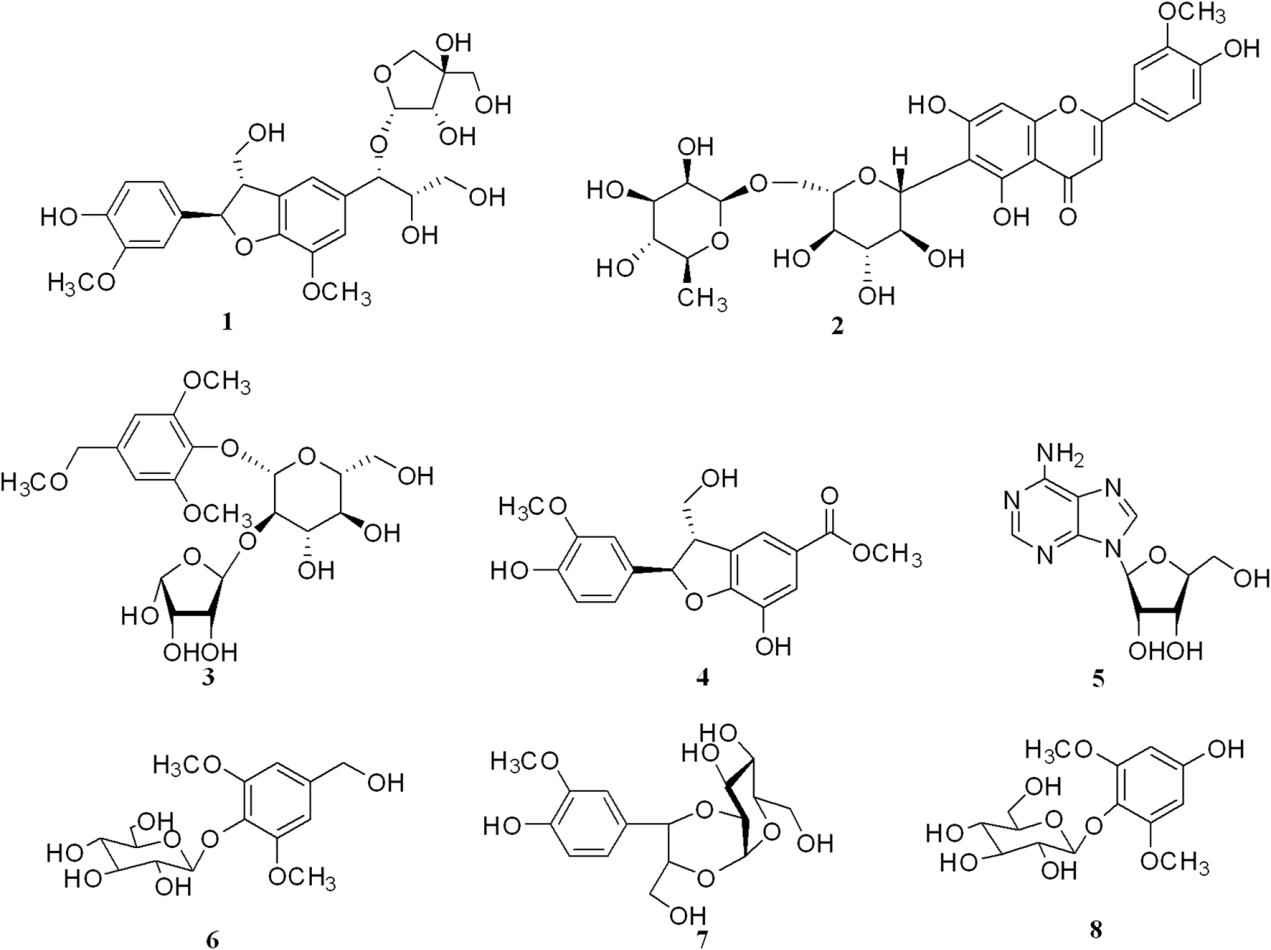
The chemical structures of compounds **1**–**8** isolated from *Tirpitzia sinensis*

In our research, the *n*-butanol extracts of the aerial part of *T. sinensis* was examined for the presence of midpolar anti-inflammatory compounds. Ultimately three new compounds (**1**–**3**), along with five known compounds (**4**–**8**), were isolated from the *n*-butanol extracts for the first time (Fig. 1). Only the nucleoside adenosine (**5**) has been reported previously in the genus *Tirpitzia*. We herein report the isolation and structural elucidation of three new compounds, as well as the results of a pharmacophore-based parallel screening approach to predict their potential bioactivity and pharmacological targets. Furthermore, based upon the in silico results of these new compounds, potential targets with reported effects on neuroinflammation or Alzheimer disease, such as JAK2 [16] and MAPK14 [17, 18] have been identified. Therefore, the BV-2 cell lines induced by LPS were chosen for the NO inhibition assay of the new compounds.

## Materials and methods

### General experimental procedures

HR-ESI-TOF-MS data were taken on a LCT premier XE TOF mass spectrometer (Waters Corp., USA). 1D NMR spectra were recorded with a Bruker Advance DRX 600 instrument operating at 600 MHz for ^1^H NMR and at 151 MHz for ^13^C NMR, and 2D NMR spectra were obtained with the standard Bruker software (Bruker Corp., Switzerland). IR data were tested by a Bruker VERTEX 70 spectrophotometer. Jasco P2000 (Jasco International Co., Ltd., Japan) polarimeter was used to measure optical rotations. Medium-pressure liquid chromatography (MPLC) separation was performed using a flash chromatography system equipped with pump module C-605, control unit C-620, fraction collector C-660, RI detector and UV photometer C-635 (Büchi Corp., Switzerland). Recycling high-performance liquid chromatography purifications were performed with a JAI LC-9110 NEXT (Japan Analytical Industry Co., Ltd, Japan). High-speed counter-current chromatography (HSCCC) separations were conducted with a semi-preparative TBE-300A and a preparative TBE-300B HSCCC (Shanghai Tauto Biotechnique Co. Ltd., China). Column chromatography was performed on silica gel (100–200 μm mesh, 300–400 μm mesh, Qingdao Marine Chemical Inc., China), D101 macroporous resin (Qingdao Marine Chemical Inc., China), ODS (YMC, Japan) and Sephadex LH-20 (GE Healthcare Bio-Sciences AB, USA). Preparative TLC plates with silica gel GF_254_ (Yantai Institute of Chemical Industry, China) was used to detect fractions and compounds by observing under UV (254 nm) and spraying with 10% H_2_SO_4_ in EtOH, heated until the coloration developed. MQX-200 photometer (Bio-Tek. Instrument Inc., UAS) was used to read in vitro results.

### Plant material

The aerial part of *T. sinensis* were collected from Jingxi County (Guangxi Zhuang Autonomous Region, China) in June 2012, and identified by Dr. Chunlin Long (Minzu University of China). A voucher specimen (No. TS-062012) was deposited at the Laboratory of Ethnobotany, Minzu University of China.

### Extraction and isolation

The air-dried aerial part of *T. sinensis* (5 kg) was powdered and extracted with 95% EtOH under reflux (3 × 15 L, each for 3 h). The crude extracts were concentrated to give a residue, which was dissolved in H_2_O and successively partitioned with petroleum ether, EtOAc, and *n*-BuOH. Subsequently, the *n*-BuOH extracts (30 g) was run over a macroporous resin (D101) column eluting with EtOH in H_2_O (30%, 50%, 70% and 95%, each 10 L), fractions A (8.3 g), B (7.2 g), C (5.6 g) and D (3.5 g) were obtained, respectively.

Fraction A was separated by the MPLC, equipped ODS column (230 × 26 mm), and eluted with a gradient of MeOH in H_2_O (5–100%) to obtain five fractions (Fr. A1–Fr. A5), based on TLC profiles. Fr. A3 was subjected to Sephadex LH-20 gel column (2.5 × 250 cm) in MeOH/H_2_O (v/v = 1:1) to give compound **5** (2.8 mg). Subfractions of Fr. A3 were purified by preparative recycling-HPLC and eluted with MeOH, yielded compound **3** (6.6 mg) from Fr. A3-18–20, compound **6** (29.4 mg) from Fr. A3-23–27, compound **7** (10.9 mg) and compound **8** (22.4 mg) from Fr. A3-30.

Fraction B was recombined as six parts (Fr. B1–Fr. B6), based on TLC profiles. Fr. B3 was subjected to MPLC with a MeOH and H_2_O mixture (5–100%) as the eluent. Then, the 50% MeOH/H_2_O eluate was further separated by Sephadex LH-20 gel column in MeOH to give to Fr. B3-7, which through Sephadex LH-20 gel column in MeOH again and obtained Fr. B3-7_(28)_. Compound **4** (3.2 mg) was obtained from Fr. B3-7_(28)_ following preparative recycling-HPLC purification with 80% MeOH/H_2_O. Fr. B4 was separated on the HSCCC (condition: EtOAc–*n*BuOH–H_2_O (1:4:5), 950 rad/min, 2 mL/min, 28 °C) to yield 20 fractions (Fr. B4-1 to Fr. B4-20) collecting 50 mL each fraction. Fr. B4-3–5 was subjected to silica gel column chromatography (100 × 1.5 cm, 200–300 μm mesh) eluted with CHCl_3_–MeOH gradient (v/v = 50:1, 30:1, 10:1, 5:1, 3:1, 1:1) to give 40 fractions (Fr. B4-3–5_(1)_ to Fr. B4-3–5_(40)_) collecting 10 mL each fraction. Fr. B4-3–5_(21–24)_ and Fr. B4-3–5_(33–36)_ was purified by preparative recycling-HPLC eluted with MeOH to give compound **1** (8.9 mg) and compound **2** (5.4 mg), respectively.

*Tirpitin* (***1***). Yellowish oily liquid: $[\upalpha]_{\rm D}^{25}$-12.3° (*c* 0.25, CH_3_OH); IR (KBr): 3347, 2945, 2831, 1452, 1030 cm^−1^; ^1^H NMR (600 MHz, CD_3_OD): see Table 1; ^13^C NMR (151 MHz, CD_3_OD): see Table 1; HR-TOF-ESI-MS (negative-ion mode) *m/z*: 547.1873 [M+Na]^+^ (Calcd. for C_25_H_32_O_12_Na: 547.1880).

**Table 1:** NMR data of the new compounds (**1**–**3**) isolated from *Tirpitzia sinensis* (δ in ppm, ^1^H NMR 600 MHz; ^13^C NMR 151 MHz)

Position	Compound 1 (in CD_3_OD)		Compound 2 (in CD_3_OD)		Compound 3 (in DMSO)	
	^13^C NMR	^1^H NMR	^13^C NMR	^1^H NMR	^13^C NMR	^1^H NMR
1	134.8	–			132.9	–
2	110.7	6.94 (1H, s, overlapped)	166.2	–	152.7	–
3	149.3	–	104.1	6.65 (1H, s)	104.5	6.61 (1H, s)
4	147.7	–	184.4	–	138.2	–
5	116.3	6.76 (1H, d, 8.1)	164.9	–	104.5	6.61 (1H, s)
6	119.9	6.83 (1H, dd, 8.1, 1.3)	110.5	–	152.7	–
7	89.3	5.53 (1H, d, 6.2)	166.2	–	62.9	4.40 (2H, d,4.2)
8	55.5	3.52 (1H, dt, 12.6, 5.3)	95.6	6.53 (1H, s)	56.2	3.37 (3H, s)
9	65.0	3.78 (1H, m); 3.83 (1H, m)	158.7	–	56.2	3.73 (3H, s)
10	56.6	3.81 (3H, s)	105.4	–	56.2	3.73 (3H, s)
11					108.5	5.32 (1H, d, 1.1)
12					74.0	3.51 (1H, d, 9.2)
13					76.6	3.79 (1H, d, 3.4)
14					79.3	5.19 (1H, m)
1′	136.9	–	123.6	–	100.7	4.93 (1H, d, 7.3)
2′	112.7	6.93 (1H, s, overlapped)	110.5	7.48 (1H, d, 8.1)	77.1	3.46 (1H, m)
3′	145.4	–	149.5	–	70.1	3.46 (1H, m)
4′	149.1	–	152.3	–	64.6	3.19 (1H, dq, 10.8, 3.9)
5′	129.9	–	116.7	6.94 (1H, d, 8.3)	76.8	3.57 (1H, ddd, 11.7, 4.6, 2.3)
6′	117.0	6.92 (1H, s)	121.5	7.51 (1H, dd, 8.4, 1.4)	61.0	3.86 (1H, d, 9.3); 3.02 m
7′	75.6	4.62 (1H, d, 6.2)	56.3	3.96 (3H, s)		
8′	76.1	3.83 (1H, m)				
9′	78.1	3.52 (1H, dt, 12.6, 6.2); 3.92 (1H, d, 2.5)				
10′	56.9	3.88 (3H, s)				
1′′	111.1	4.88 (1H, d, 2.6)	73.5	4.95 (1H, d, 11.3)		
2′′	70.7	3.46 (1H, dd, 10.4, 4.2)	72.1	3.58 (1H, m)		
3′′	80.6	–	82.5	3.42 (1H, m)		
4′′	75.1	3.71 (1H, d, 3.6); 3.95 (1H, d, 5.5)	81.1	3.46 (1H, m)		
5′′	65.5	3.58 (2H, d, 2.9)	101.9	3.73 (1H, m)		
6′′			69.8	3.89 (1H, d, 1.7); 3.87 (1H, m)		
1′′′			102.4	5.23 (1H, s)		
2′′′			72.1	3.87 (1H, m)		
3′′′			72.5	3.12 (1H, t, 9.3)		
4′′′			69.5	2.57 (1H, dd, 9.3, 6.2)		
5′′′			71.8	3.41 (1H, m)		
6′′′			17.2	0.75 (1H, d, overlapped)		

*Tirpitzoside* (***2***). Yellowish oily liquid: $[\upalpha]_{\rm D}^{25}$-36.2° (*c* 0.15, CH_3_OH); IR (KBr): 3348, 2945, 2833, 1451, 1031 cm^−1^; ^1^H NMR (600 MHz, CD_3_OD): see Table 1; ^13^C NMR (151 MHz, CD_3_OD): see Table 1; HR-TOF-ESI-MS (positive-ion mode) *m/z*: 631.1693 [M+Na]^+^ (Calcd. for C_28_H_32_O_15_Na: 631.1639).

*Tirpitziol* (***3***). Yellowish oily liquid: $[\upalpha]_{\rm D}^{25}$-86.4º (*c* 0.20, CH_3_OH); IR (KBr): 3363, 2946, 2833, 1031 cm^−1^; ^1^H NMR (600 MHz, DMSO): see Table 1; ^13^C NMR (151 MHz, CD_3_OD): see Table 1; HR-TOF-ESI-MS (positive-ion mode) *m/z*: 501.1586 [M+Na]^+^ (Calcd. for C_20_H_30_O_13_Na: 501.1584).

### In silico target fishing for new compounds

**Fig. 2:**
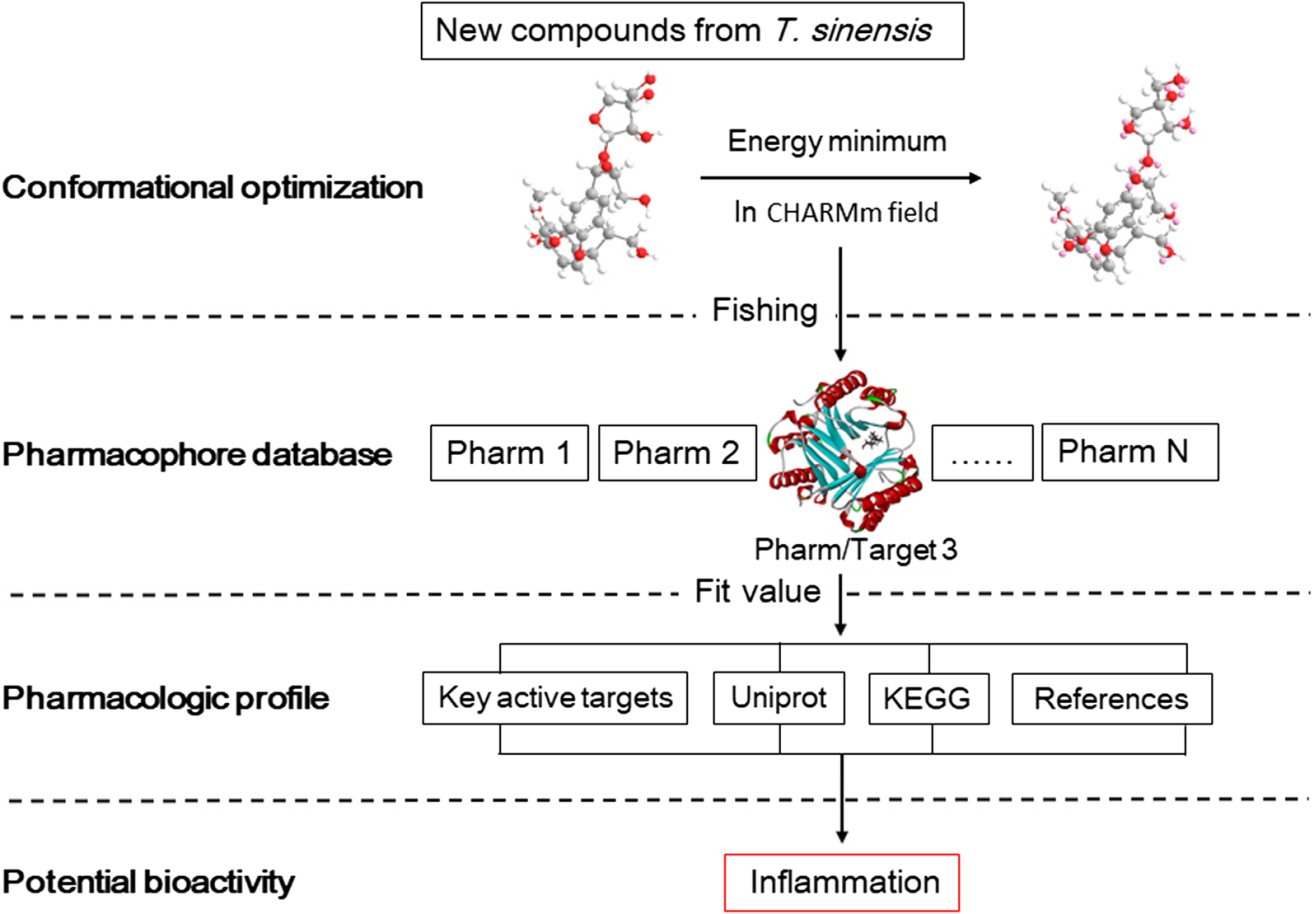
The workflow of target fishing in silico

Pharmacophore-based parallel screening of three new compounds isolated from *T. sinensis* were implemented by Discovery Studio 4.0 software (DS) as the following two steps: (1) Conformational optimization of tested compounds. The two-dimensional (2D) structures of compounds drawn by ChemDraw were translated to three-dimensional (3D) structures in DS. Small molecules protocol with Full Minimization algorithm was used for the 3D conformations of energy minimum of compounds. These small molecules subjected to training set based on CHARMm force field. (2) Pharmacophore-based parallel screening based on Ligand Profiler procedure. Optimized 3D conformations were submitted to Ligand Profiler procedure belonged to Pharmacophore Search module, and set the following conditions: ‘Input PharmaDB Pharmacophores’ was set to ALL and SHAPE was selected; ‘Conformation Generation’ set to FAST; TRUE was selected in the ‘Save Conformations’; then all other parameters were set to their default values in DS. After the above operations, Ligand Profiler allows testing three compounds simultaneous against all of pharmacophore models without omitting any features. Moreover, the resulting lists will display the pharmacophore hits according to fit value and the corresponding target ID, KEGG ID and target type, which help to estimate the potential bioactivities of the three new compounds. The overall strategy of target fishing in silico was shown in Fig. 2.

### Cell culture and NO inhibitory assay

BV-2 cells, provided by the Cell Culture Center at the Institute of Basic Medical Sciences, Chinese Academy of Medical Sciences, were cultured in Dulbecco’s modified Eagle’s medium (DMEM/F-12) (Gibco Co., NY, USA) supplemented with 10% fetal bovine serum, 100 µg/mL streptomycin, and 100 U/mL penicillin. Cells were initially grown in 96-well plate (Corning Co. NY, USA) (2 × 10^5^ cells/well) and pre-treated 24 h later with compounds and positive control at concentration of 10 µM, 1 µM and 0.1 µM, respectively. The compounds and curcumin was dissolved in DMSO, and the final concentration of DMSO in medium was less than 0.1%. Cells were stimulated 1 h later with LPS 300 ng/mL and then continue to incubate for 24 h. Next, 100 µL supernatant of culture medium from the sample was mixed with an equal volume of Griess reagent (Sigma-Aldrich, MO, USA) (1% sulphanilamide in 5% phosphoric acid and 0.1% *N*-naphthyl-ethylenediamine dihydrochloride) in a 96-well plate, incubated at room temperature for 10 min. After incubation the absorbance was determined spectrophotometrically at 540 nm. Fresh culture medium was used for blank-reading in all experiments, and the positive control was curcumin [19]. The amount of NO was calculated with reference to a sodium nitrite standard curve freshly prepared in culture medium. Cell viability controls were used to ensure that the observed NO reduction was not due to any cytotoxicity of the compounds we tested.

### Statistical analysis

The inhibition percentage was calculated by the Eq. (1):

1 $$\text{H} = \left( {\text{ODc} - \text{ODs}} \right)/\left( {\text{ODc} - \text{ODb}} \right) \times 100$$

In this equation, H is the inhibition percentage (%), ODc, ODs and ODb are the absorbance of control, sample and blank at 540 nm, respectively. The concentrations of compounds that gave 50% inhibiting potential (IC_50_, µM) was calculated by modified Kou type Eq. (2):

2 $$\text{lgIC}_{50} = \text{X}_{\text{m}} - \text{I}\left( {\text{P} - \left( {3 - {\mathbf{P}}_{\text{m}} - {\mathbf{P}}_{\text{n}} } \right)/4} \right)$$

where X_m_ is the lg of the maximum dose; P is the sum of positive response rate; P_m_ is the largest positive response rate; P_n_ is the smallest positive response rate; I is the lg of the (maximum dose/adjacent dose) [20]. The data analyses were performed with Microsoft Excel 2013. The assays were tested in triplicate, and results were present as mean ± SD.

## Results and discussion

### Structure elucidation

**Fig. 3:**
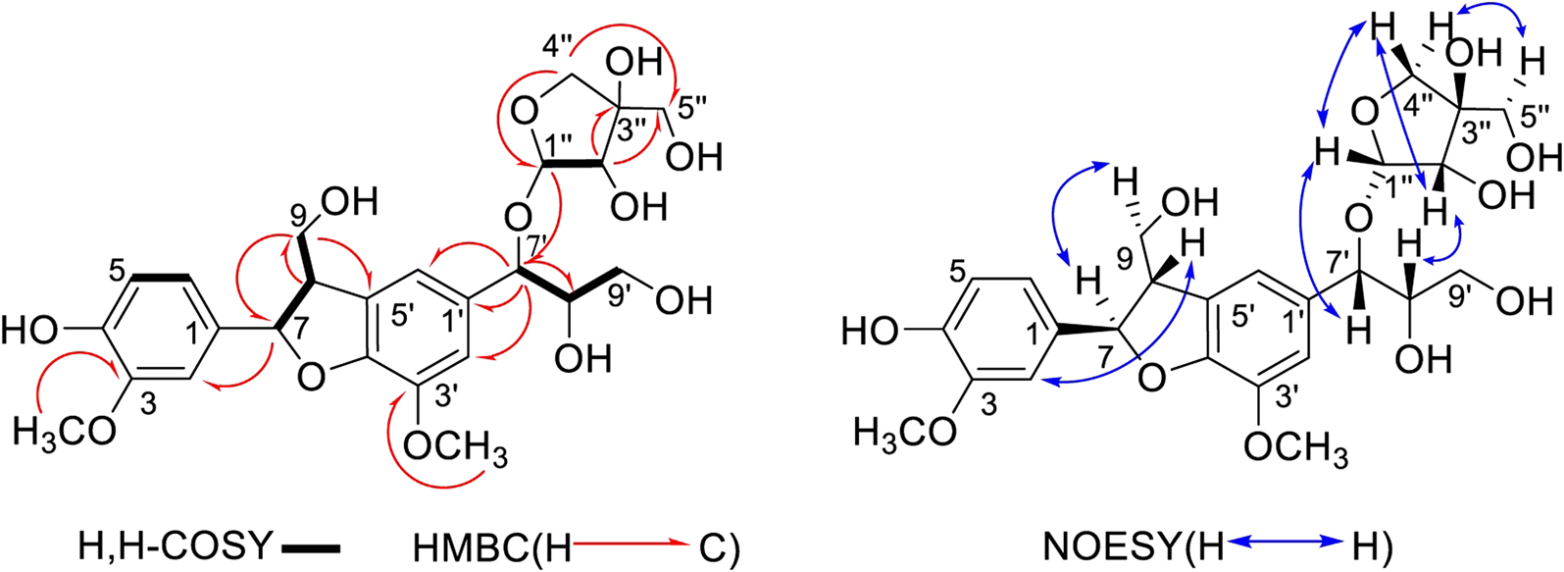
Key NMR correlations of compound 1

Tirpitin (**1**) was obtained as a yellowish oily liquid. The HR-TOF-ESI-MS spectrum of **1** supported a molecular formula of C_25_H_32_O_12_ (*m/z* 547.1873 [M+Na]^+^, calcd. for C_25_H_32_O_12_Na, 547.1880), indicating 10 degrees of unsaturation (all spectra were shown in Additional file 1: Fig. S1). The IR spectrum showed absorptions for hydroxyl group (3347 cm^−1^), two methoxy groups (2945 cm^−1^ and 2832 cm^−1^) and an aromatic ring (1451 cm^−1^). The ^1^H NMR spectrum (Table 1) exhibited a ABX system at *δ*_H_ 6.94 (1H, s, overlapped, H-2), 6.76 (1H, d, *J* = 8.1 Hz, H-5) and 6.83 (1H, dd, *J* = 8.1, 1.3 Hz, H-6), as well as another AB system at 6.93 (1H, s, overlapped, H-2′) and 6.92 (1H, s, H-6′), indicating there are a 1,3,4,5-tetrasubstituted and a 1,3,4-trisubstituted aromatic ring. An anomeric proton at *δ*_H_ 4.88 (1H, d, *J* = 2.6 Hz) correlated with the carbon at *δ*_C_ 111.1 in HSQC spectrum, which indicated that one sugar moiety may be connected via an *O*-linkage [*δ*_C_ 111.1 (C-1′′)]. All the proton signals of the sugar unit were assigned by HSQC, ^1^H–^1^H COSY and HMBC spectra. Furthermore, the presence of a doublet at *δ*_H_ 5.53 (1H, d, *J* = 6.2 Hz, H-7) and three characteristic carbon signals at *δ*_C_ 89.3 (C-7), 55.5 (C-8) and 65.0 (C-9) indicated that compound **1** belongs to a group of dihydrobenzofuran-type lignans, which was confirmed by ^1^H–^1^H COSY correlations of H-7/H-8/H-9 (Fig. 3). In the ^1^H–^1^H COSY spectrum, H-5/H-6, H-7′/H-8′/H-9′ and H-1′′/H-2′′ correlations were observed. The ^1^H NMR spectrum displayed two methoxy protons signals at *δ*_H_ 3.87 (3H, s) and 3.81 (3H, s).

The ^13^C NMR data showed 25 carbon signals of compound **1**, including 2 methyls, 4 methylenes, 11 methines, and 8 quaternary carbons in the DEPT experiment. The presence of an apiofuranosyl unit was suggested by two oxygenated methylene carbons at *δ*_C_ 75.1 and 65.6, one oxygenated methine carbon at *δ*_C_ 70.7, one quaternary carbon at *δ*_C_ 80.6 and one anomeric carbon at *δ*_C_ 111.1 (Table 1), which were identified as α configuration based on the *J* value-coupling constant of H-1′′ (*J *= 2.6 Hz) [21]. The HMBC correlations from proton at H-7′ (*δ*_H_ 4.62) to the carbons C-1′ (*δ*_C_ 136.9), C-2′ (*δ*_C_ 112.7), and C-6′ (*δ*_C_ 117.0) indicated that the group of C-7′/C-8′/C-9′ was attached to C-1′ of lignan skeleton (Fig. 3). Further, an HMBC correlation between the anomeric proton *δ*_H_ 4.88 and C-7′ (*δ*_C_ 75.6) indicated the apiofuranosyl moiety was connected to C-7′ of the lignan aglycone.

When comparing the NMR data with (7*R*,8*S*,7′*R*,8′*S*)-1′-trihydroxypropyl-3′-methoxy-8-hydroxymethyl-7-(4-hydroxy-3-methoxyphenyl)-7,8-dihydrobenzofuran lignan (prinsepin A) isolated from the fruit of *Prinsepia uniflora* Batal [22], the data for compound **1** were very similar to prinsepin A except that the apiose moiety in **1** has replaced the hydroxyl group in prinsepin A at C-7′, suggesting **1** should show the same stereochemical assignments as prinsepin A at C-7, C-8, C-7′ and C-8′. The large *J* value (6.2 Hz) between H-7 and H-8, and H-7′ and H-8′ indicated that there are both *threo* diastereomers [23]. These relative stereochemical assignments for **1** can be also supported by its NOESY spectrum, the correlations of H-7/H-9α (*δ*_H_ 3.78) and H-8/H-2 indicated that H-7 was *α* orientation, and H-8 was *β* orientation. The relative stereochemical assignments of the apiose moiety was also confirmed by the NOESY correlations of H-1′′/H-7′, H-1′′/H-4′′β (*δ*_H_ 3.71), and H-4′′α (*δ*_H_ 3.95)/H-5′′ (Fig. 3). The NOESY correlations of H-1′′/H-7′, H-2′′/H-8′, and H-4′′α/H-5′′ also indicated that H-7′, H-8′ and the hydroxyl attached on C-3′′ are *β* orientation. Based on these analyses described above, the structure **1** was elucidated as (7R*,8S*,7′R*,8′S*)-1′-trihydroxypropyl-3′-methoxy-8-hydroxymethyl-7-(4-hydroxy-3-methoxyphenyl)-7,8-dihydrobenzofuran lignan-7′-*β*-apioside as a new compound, named tirpitzin.

**Fig. 4:**
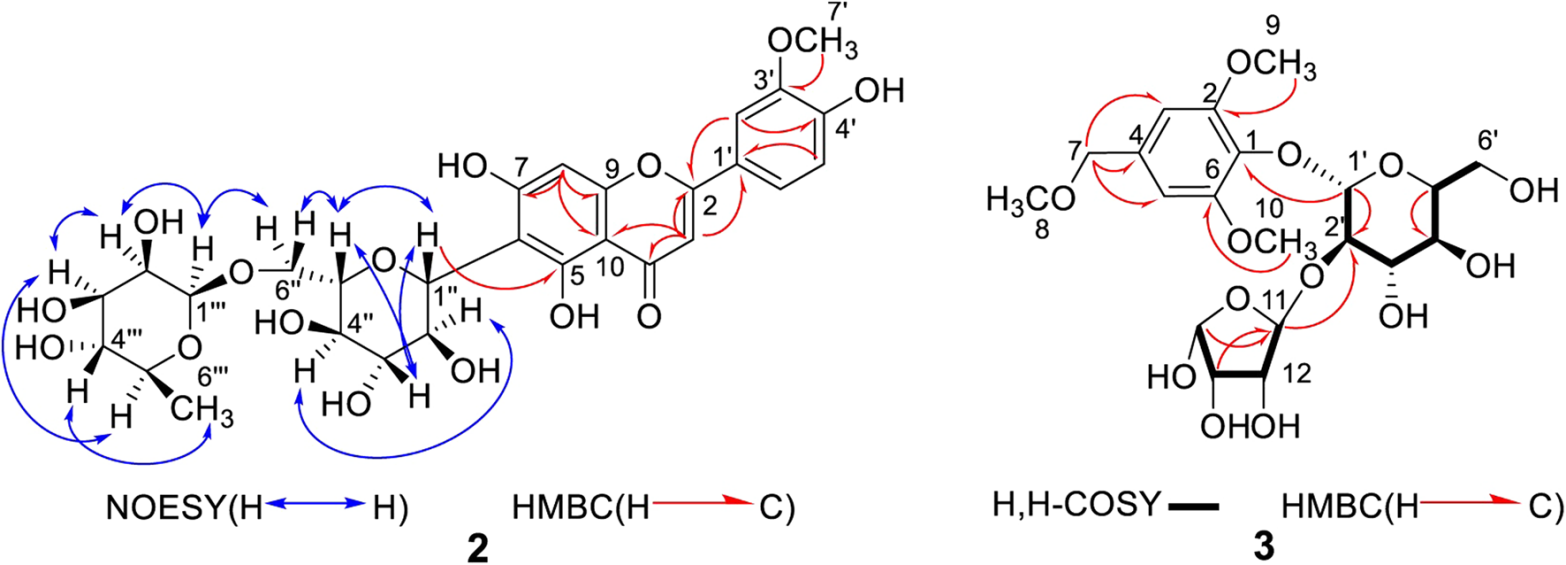
Key NMR correlations of compounds 2 and 3

The molecular formula of **2** was established as C_28_H_32_O_15_ (*m/z* 631.1693 [M+Na]^+^, calcd. for C_28_H_32_O_15_Na, 631.1639) by HR-TOF-ESI-MS in the positive spectrum (all spectra were shown in Additional file 1: Fig. S2). The IR spectrum of **2** showed hydroxyl groups, methoxy groups and aromatic rings respectively at 3347, 2945, 2832, 1451 and 1030 cm^−1^. The ^1^H NMR spectrum (Table 1) of **2** revealed an ABX system at *δ*_H_ 7.51 (1H, dd, *J* = 8.4, 1.4 Hz, H-6′), 7.48 (1H, d, *J* = 1.4 Hz, H-2′) and 6.94 (1H, d, *J* = 8.3 Hz, H-5′), and an olefinic signal at *δ*_H_ 6.65 assigned to H-3 position, which indicated it is a flavone skeleton [21]. Additionally, one aromatic singlet signal at *δ*_H_ 6.53, one methoxyl signal at *δ*_H_ 3.96 (3H, s), one doublet methyl signal at *δ*_H_ 0.75 (3H, d, overlapped), two anomeric proton signals at *δ*_H_ 5.23 (1H, s) and *δ*_H_ 4.95 (1H, d, *J* = 11.3 Hz) were observed as well. In the HSQC spectrum, *δ*_H_ 5.23 (1H, s) correlated with the carbon signal at *δ*_C_ 102.4 (C-1′) indicating that it is connected through an *O*-linkage, while the other anomeric proton *δ*_H_ 4.95 correlated with C-1′′ (*δ*_C_ 73.5), suggesting it is connected to the flavone skeleton by a C-linkage [24]. The doublet methyl signal at *δ*_H_ 0.75 (3H, d, overlapped) suggested a rhamnosyl residue in **2**, which was further confirmed by the group of carbon signals at *δ*_C_ 102.4, 72.5, 72.1, 71.8, 69.5 and 17.2. The other sugar moiety was a glucopyranosyl unit for the group of carbon signals at *δ*_C_ 101.9, 82.5, 81.1, 73.5, 72.1 and 69.8. A total of 28 carbon signals of **2** was exhibited from the analysis of ^13^C NMR, HSQC and HMBC spectra, which corresponds to one flavonoid moiety, one glucopyranosyl unit, one rhamnosyl unit and one methoxy carbon. The last aromatic proton signal at *δ*_H_ 6.53 (1H, s) correlates to the C-8 position of A ring in the HSQC spectrum, and thereby indicating that there are two hydroxy-substituted at C-5 and C-7 of the A ring. In the HMBC spectrum, the correlations from H-7′ to C-3′, H-2′ to C-4′ and C-2, and H-5′ to C-1′ respectively, establishing the *ortho*-substitution of B ring at C-3′ and C-4′, and methoxy located at C-3′ and hydroxy at C-4′ (*δ*152.3) for the downfield shift of C-4′ (Fig. 4). The HMBC correlations from H-3 to C-1′, C-2, C-4, C-10, H-8 to C-7, C-9, C-10, and H-1′′ to C-5, confirmed that the sugar unit was attached at C-6 of the flavone aglycon.

The glucopyranosyl moiety was identified as *β*-d-configuration based on the coupling constant of H-1′′ (*J *= 11.3 Hz) [25], and further supported by the NOESY spectrum. The NOESY cross-peaks (Fig. 4) between H-1′′ and H-3′′, H-1′′ and H-5′′, H-3′′ and H-5′′, H-2′′ and H-4′′, indicated that H-1′′, H-3′′ and H-5′′ were axial and *β*-glucose. The NOESY correlations for H-1′′′/H-2′′′, H-2′′′/H-3′′′, H-3′′′/H-5′′′, and H-4′′′/H-6′′′ indicated that H-1′′, H-3′′ and H-5′′ were *α*-oriented, and H-4′′′, H-6′′′ were *β*-oriented. Furthermore, the obvious downfield shift of glucose methylene carbon (C-6′′) at *δ*_C_ 69.8 indicated the effect of glycosidation, which suggested anomeric proton of rhamnose *δ*_H_ 5.23 (s) was located at C-6′′ hydroxyl group of the glucosyl moiety with α configuration and 6 → 1 sequence linkage [21, 26], also supported by the NOESY correlations of H-5′′/H-6′′β (*δ*_H_ 3.87) and H-1′′′/H-6′′α (*δ*_H_ 3.89). On the basis of these combined data, compound **2** was identified as 3′-*O*-methylluteolin-6-*C*-[1′′-*β*-d-glucopyranosyl (6 → 1)-*α*-l-rhamnoside]. Comparison of the NMR data of **2** and known compounds, chrysoeriol-7-*O*-[*α*-l-rhamnopyranosyl-(1 → 6)-*β*-d-glucopyranoside] [27], showed similarity in flavones aglycon except for significant differences in the sugar portion. Therefore, compound **2** was established a new flavone, named tirpitzoside.

The HR-TOF-ESI-MS spectrum of **3** exhibited a molecular formula of C_20_H_30_O_13_ (*m/z* 501.1586 [M+Na]^+^ calcd. for C_20_H_30_O_13_Na, 501.1584), displaying 6 degrees of unsaturation (all spectra were shown in Additional file 1: Fig. S3). The IR spectrum showed absorptions for hydroxyl group (3362 cm^−1^), methoxy groups (2945 cm^−1^) and aromatic ring (1030 cm^−1^). The ^1^H NMR spectrum of **3** indicated the existence of a 1,2,4,6-tetrasubstituted benzene ring from two singlets aromatic protons at *δ*_H_ 6.6, three methoxy signals at *δ*_H_ 3.73 (6H, s), 3.37 (3H, m). An anomeric proton signal at *δ*_H_ 4.97 (1H, d, *J* = 7.3 Hz) was correlated to the carbon signal at *δ*_C_ 100.7 (C-1′) in HSQC spectrum. Moreover, the ^1^H-^1^H COSY and HSQC spectra (Fig. 4) supported the assignments of H-2′ (*δ*_H_ 3.75), H-3′ (*δ*_H_ 3.82), H-4′ (*δ*_H_ 3.41), H-5′ (*δ*_H_ 4.19) and H-6′ (*δ*_H_ 3.54 and *δ*_H_ 3.35), respectively. The ^1^H–^1^H COSY displayed the correlation of H-11/H-12/H-13/H-14 as well. In the ^13^C NMR spectrum, the signals at δc 152.6, 104.5 and 56.2 were considered as three overlapping carbon signals due to their peak intensity, and were assigned as C-2/C-6, C-3/C-5, and C-9/C-10, respectively. The carbon signals (Table 1) showed a glucopyranosyl group (*δ*c 100.7, 77.1, 76.8, 70.1, 64.6 and 61.0), three methoxy group (*δ*c 56.2, 56.2 and 56.2), an aromatic ring (*δ*c 152.6, 152.6, 138.8, 132.4, 104.5 and 104.5), and an epoxy butanol unit (*δ*c 108.5, 79.3, 76.6, and 74.0), corresponding to the ^1^H–^1^H COSY correlations of H-11/H-12/H-13/H-14.

The presence of a methoxymethyl fragment was supported by the *J* value and *δ*_H_ of H-8 (d, *δ*_H_ 3.37), H-7 (dd, *J* = 8.3, 1.6 Hz, *δ*_H_ 4.40), as well as this fragment was attached at C-4 according to HMBC correlations from the proton at *δ*_H_ 4.40 (H-7) to the carbon signals at *δ*c 104.5 (C-3), 138.2 (C-4) and 104.5 (C-5) (Fig. 4). Another two methoxy groups were assigned to C-2 and C-6 (*δ*c 152.7) for HMBC correlations of *δ*_H_ 3.73 (H-9) to *δ*c 152.7 (C-2) and *δ*_H_ 3.73 (H-10) to *δ*c 152.7 (C-6), respectively. The sugar residue was identified as a *β*-d-glucopyranosyl configuration for the large ^*3*^*J* value (7.3 Hz) of the anomeric proton [27], and its linkage was established by the HMBC cross-peaks between *δ*_H_ 4.97 (H-1′) and *δ*c 132.9 (C-1). The significant downfield shift of H-11 (*δ*_H_ 5.32) and C-11 (*δ* 108.5) indicated the epoxy butanol unit was attached to C-2′ (*δ*c 77.1), which was also confirmed by HMBC correlations from H-11 to C-2′. NMR data comparisons of **3** with known compounds, 4-methoxy-2,5-dimethylphenyl-*α*-l-arabinofuranosyl-(1 → 6)-*β*-d-glucopyranoside [28], 3,4-dimethoxyphenyl-2-*O*-(3-*O*-methyl-*α*-l-rhamnopyranosyl)-*β*-d-glucopyranoside [29], indicated that **3** was similar to these compounds except for the methoxymethyl group and epoxy butanol group in **3**. Thus, **3** was identified as (2,6-dimethoxy-4-(methoxymethyl)-phenoxy)-tetrahydro-3′,4′-dihydroxy-5-(hydroxymethyl)-pyran-2′-yloxy)-tetrahydrofuran-12,13,14-triol, and given the common name tirpitziol.

Using NMR and MS data, the other known compounds were identified as (2*S*,3*R*)-methyl-7-hydroxy-2-(4-hydroxy-3-methoxyphenyl)-3-(hydroxymethyl)-2,3-dihydrobenzofuran-5-carboxylate (**4**) [30], adenosine (**5**) [31, 32], 3,5-dimethoxy-benzyl alcohol 4-*O*-*β*-d-glucopyranoside (**6**) [33], 3-methoxy-4-hydroxy-phenylpropane-7,8-(2′,1′-*O*-*β*-d-glucopyranosyl)-7,8,9-triol (**7**) [34, 35] and 2,6-dimethoxy-4-hydroquinone-1-*O*-*β*-d-glucopyranoside (**8**) [36].

### Bioactive screening based on target fishing

Pharmacophore-based parallel screening was conducted in silico with a program that examines each testing compound against all pharmacophore models, and then a result will be generated indicating the number of target hits and their corresponding fit value. The fit value is computed to evaluate how well the compound maps the chemical function-based features of the pharmacophore. Furthermore, the closer the fit value is to the integer 1, the higher confidence-level this value has. Therefore, we will focus on the biological properties of the predicted targets which have a higher fit value. Focusing on these targets and learning and understanding their biological roles could assist us to evaluate the potential bioactivities of the isolated new compounds and guide us to conduct related bioactive experiments. In this study, the parallel screening report found that compounds **1**–**3** could each fit multiple pharmacophore models (3, 146 and 218, respectively).

Compound **1** matched with three pharmacophore models, but only one of these had a fit value > 0.5. The fit value for the pharmacophore models (ID 3pp0) of **1** is 0.5067, and this pharmacophore corresponding receptor tyrosine-protein kinase erbB2 as shown in Table 2. It has been reported that when erbB2 was activated by heregulin-α in cells in a wound, the epithelial integrity will be restored more quickly [37]. In addition, erbB2 was also reported to mediate the interleukin-6 (a regulator of immune and inflammatory responses) for the activation of mitogen-activated protein kinase [38].

**Table 2:** Target fishing results of the new compounds (1–3) isolated from *Tirpitzia sinensis*

Compound	Pharma-ID	Fit value	Gene-name	Uniprot-AC	KEGG-identify	Target-class	Target-class A
**1**	3pp0	0.506772	ERBB2_HUMAN	P04626	K05083	Receptor tyr kinase	ERBB2
**2**	2nry	0.858086	IRAK4_HUMAN	Q9NWZ3	K04733	Interleukin-1 receptors	IRAK4
	3ac3	0.798580	LCK_HUMAN	P06239	K05856	Tyr protein kinases	LCK
	3io7	0.769459	JAK2_HUMAN	O60674	K04447	Tyr protein kinases	JAK2
**3**	3zya	0.916562	MK14_HUMAN	Q16539	K04441	Ser/Thr protein kinases	MAPK14
	3ehx	0.816364	MMP12_HUMAN	P39900	K01413	Zinc-dependent endopeptidases	MMP-12

ERBB2 means receptor tyrosine-protein kinase erbB-2; IRAK4 means interleukin-1 receptor-associated kinase 4; LCK means lymphocyte specific kinase; JAK2 means Janus kinase 2; MAPK14 means mitogen-activated protein kinase 14; MMP-12 means matrix metalloproteinase 12

As for **2**, we focused our studies on the following human pharmacological targets, interleukin-1-receptor-associated kinases 4 (IRAK4, pharmacophore models 2nry), tyrosine kinase (LcK, pharmacophore models 3ac3), and Janus kinase 2 (JAK2, pharmacophore models 3io7), with fit values of 0.8581, 0.7986, and 0.7695, respectively (Table 2). IRAK4 has been reported to lead to the increase of MAPK signaling pathway NF-κB protein, and IκB kinase, and subsequently, the activated NF-κB promotes the expression of the downstream target inflammatory cytokines [39]. In human pericytes, the inhibition of IRAK4 could significantly reduce myeloid differentiation primary response gene 88 (MyD88)-mediated inflammatory responses to kidney damage-associated molecular patterns (DAMPs) characterized by reduction in *IL6* and *CCL2* expression [40]. IRAK4 is known to be important in normal inflammatory reactions derived from nonbacterial or bacterial infections [41]. LcK, a tyrosine kinase, is not only a positive regulator of the mitochondrial apoptosis pathway [42], but also plays a critical role in T cell activation [43], e.g. effects in development and activation of T-cells including T-cell antigen receptor phosphorylation, which led to the production of cytokines such as IL-2 and interferon gamma and causes activation and proliferation of T-lymphocytes to generate an immune response [44]. JAK2 is involved in variety of inflammatory signaling pathways, multiple physiological and pathological regulation processes [45]. The activation of JAK2 can increase expression of high-mobility group box protein 1, which promotes the release of cytokines such as TNF-α inducing the inflammatory reaction [46]. It also reported the inhibition of JAK2 can effectively block the IFNg-induced changes in microglia, suggesting that JAK2 inhibition is a potential treatment of neuroinflammation [16].

From the virtual screening results of compound **3**, we focused on two pharmacological targets, mitogen-activated protein kinase 14 (MAPK14) and matrix metalloproteinase (MMP-12), corresponding pharmacophore models 3zya (fit value 0.9166) and 3ehx (fit value 0.8164), respectively (Table 2). MAPK14 (also known as MAPK p38α) is commonly expressed in various adult tissues and can mediate cellular responses to injurious stress and immune signaling [47]. The up-regulated expression of MAPK14/p38α protein in the brain of APP (amyloid b [A4] precursor protein)-PS1 (presenillin 1) (APP-PS1) transgenic Alzheimer mouse will lead to increase autophagy and reduce amyloid pathology, which suggests that therapeutic inhibition of MAPK14 has the potential to address the autophagic defect in Alzheimer disease [17]. Additionally, MMP-12 expression has been closely linked to tissue inflammation according to its effect in matrix remodeling, and regulated inflammatory cell trafficking [48, 49]. MMP-12 inhibition exacerbates cardiac dysfunction by disrupting the CD44-HA axis to increase and prolong inflammation and reduce neutrophil apoptosis [50]. MMP-12 showed certain effects on the proliferation of corneal epithelial cells during wound healing as well [51].

Combining proper fit value and the pivotal functions of erbB2, IRAK4, LcK, JAK2, MAPK14, and MMP-12, all of these targets are related, in part, to inflammation and/or wound healing which indicate that the isolated three new compounds from *T. sinensis* may play a role in wound healing and inflammation. Based on in silico target fishing results, we have found potential biological targets for the three new compounds and summarized some of their bioactivities with regard to inflammation, and these are consistent with the traditional medicinal knowledge of *T. sinensis.*

### NO production inhibitory assay

Nitric oxide is widely accepted as an important factor of the inflammatory process [52]. NO is rapidly converted to nitrite in the presence of oxygen, the secretory activity of cells is estimated by determining nitrite concentrations after the colorimetric Griess reaction. Thus, in order to investigate whether the new compounds exhibited anti-inflammation as the predicted targets showed, we performed NO production inhibitory assay in LPS-induced BV-2 cells. Compounds **1**–**3** all showed moderate inhibitory effects (Table 3), with IC_50_ values of 14.97 ± 0.87, 26.63 ± 1.32, and 17.09 ± 2.3 μM respectively, whereas the positive control (curcumin) gave an IC_50_ value of 4.75 μM. In addition, the similar types of natural products in Greiss assay have not displayed cytotoxicity at the concentrations conducted in this studies [53–55]. The in vitro results of NO inhibition was show the interaction of new compounds with anti-inflammatory, and thereby in part explains the traditional usage of *T. sinensis* as well.

**Table 3:** NO inhibitory activity of the new compounds (1–3) from aerial part of *Tirpitzia sinensis*

Compound	Dose (μM)	NO inhibition rate (%)	IC_50_^a^ (μM)
**1**	0.1	0	14.97 ± 0.87
	1	11.97 ± 0.41	
	10	36.47 ± 2.37	
**2**	0.1	0	26.63 ± 1.32
	1	7.40 ± 1.68	
	10	20.10 ± 0.43	
**3**	0.1	3.93 ± 0.57	17.09 ± 2.3
	1	18.04 ± 1.93	
	10	23.33 ± 2.42	

^a^The concentrations of compounds that gave 50% inhibiting potential was calculated by modified Kou type equation: lgIC_50_ = X_m_ − I(P − (3 − P_m_ − P_n_)/4). Positive control (curcumin) gave an IC_50_ value of 4.75 μΜ

## Conclusions

*Tirpitzia sinensis* is an important traditional medicinal plant of the Zhuang people in Southwest China. In an attempt to validate the folk medical use of the plant, we focused our studies on its constituents and their potential bioactivities. Three new compounds (**1**–**3**), along with five known compounds, were isolated and identified from the aerial part of *T. sinensis* for the first time. The in silico results provided potential biological targets for the new compounds, and in vitro assays further demonstrated the anti-inflammatory activity. Our results, in part, help to explain the traditional usage of *T. sinensis* for treatment of wound and inflammation.

## Additional file

**Additional file 1.** The details spectra for the identification of compound **1–3**. This file includes 1D-NMR, 2D-NMR, HR-MS, IR and UV spectra of compound **1–3**.

## Abbreviations

NMR: nuclear magnetic resonance
2D-NMR: two-dimension nuclear magnetic resonance
LC/MS: liquid chromatography equipped with mass spectrometry
IR: infra-red
ERBB2: receptor tyrosine-protein kinase erbB-2
IRAK4: interleukin-1 receptor-associated kinase 4
LCK: lymphocyte specific kinase
JAK2: Janus kinase 2
MAPK14: mitogen-activated protein kinase 14
MMP-12: matrix metalloproteinase 12
NO: nitric oxide
LPS: lipopolysaccharide
HR-ESI-TOF-MS: high-resolution time of flight electrospray ionization mass spectrometry
MPLC: medium-pressure liquid chromatography
HSCCC: high-speed counter-current chromatography
TLC: thin layer chromatography
UV: ultra-violet
EtOH: ethanol
EtOAc: ethyl acetate
*n*-BuOH: *n*-butanol
MeOH: methanol
CHCl_3_: chloroform
DMSO: dimethylsulfoxide
DMEM: Dulbecco’s modified Eagle’s medium
OD: optical density
SD: standard deviation
IC50: 50% inhibiting potential concentration
HSQC: heteronuclear single-quantum correlation spectroscopy
COSY: correlation spectroscopy
HMBC: heteronuclear multiple-bond correlation spectroscopy
DEPT: distortionless enhancement by polarization transfer
NOESY: nuclear overhauser effect spectroscopy
TNF-α: tumor necrosis factor alpha
IFNg: interferon gamma
APP: amyloid b [A4] precursor protein
PS1: presenillin 1
