# Supplementary material for: Three new compounds with nitric oxide inhibitory activity from Tirpitzia sinensis, an ethnomedicinal plant from Southwest China
Source: BMC Chem. 2019 Apr 1;13(1):47. doi: 10.1186/s13065-019-0568-9 (PMC6661779; doi:10.1186/s13065-019-0568-9)

List of Additional file 1

Figure S1. Spectra of compound **1**

Figure S1-1 ^{1}H-NMR spectrum of compound **1**
Figure S1-2 ^{13}C-NMR and DEPT spectra of compound **1**
Figure S1-3 ^{1}H-^{1}H-COSY spectrum of compound **1**
Figure S1-4 HSQC spectrum of compound **1**
Figure S1-5 HMBC spectrum of compound **1**
Figure S1-6 NOESY spectrum of compound **1**
Figure S1-7 HR-LC-TOF-MS positive spectrum of compound **1**
Figure S1-8 IR spectrum of compound **1**
Figure S1-9 UV spectrum of compound **1**

Figure S2. Spectra of compound **2**

Figure S2-1 ^{1}H-NMR spectrum of compound **2**
Figure S2-2 ^{13}C-NMR and DEPT spectra of compound **2**
Figure S2-3 HSQC spectrum of compound **2**
Figure S2-4 HMBC spectrum of compound **2**
Figure S2-5 NOESY spectrum of compound **2**
Figure S2-6 HR-LC-TOF-MS positive spectrum of compound **2**
Figure S2-7 IR spectrum of compound **2**
Figure S2-8 UV spectrum of compound **2**

Figure S3. Spectra of compound **3**

Figure S3-1 ^{1}H-NMR spectrum of compound **3**
Figure S3-2 ^{13}C-NMR spectrum of compound **3**
Figure S3-3 HSQC spectrum of compound **3**
Figure S3-4 HMBC spectrum of compound **3**
Figure S3-5 ^{1}H-^{1}H COSY spectrum of compound **3**
Figure S3-6 HR-LC-TOF-MS positive spectrum of compound **3**
Figure S3-7 IR spectrum of compound **3**
Figure S3-8 UV spectrum of compound **3**

Figure S1. Spectra of compound **1**

Figure S1-1 ^{1}H-NMR spectrum of compound **1**

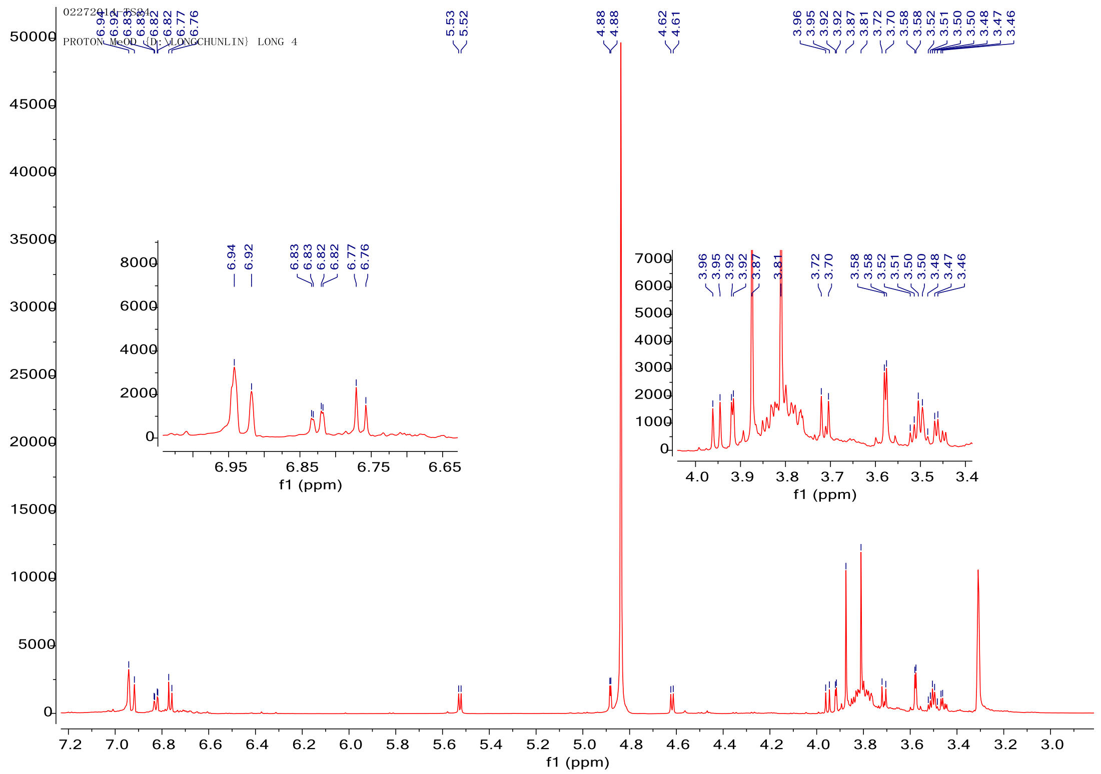

Figure S1-2 ^{13}C-NMR and DEPT spectra of compound **1**

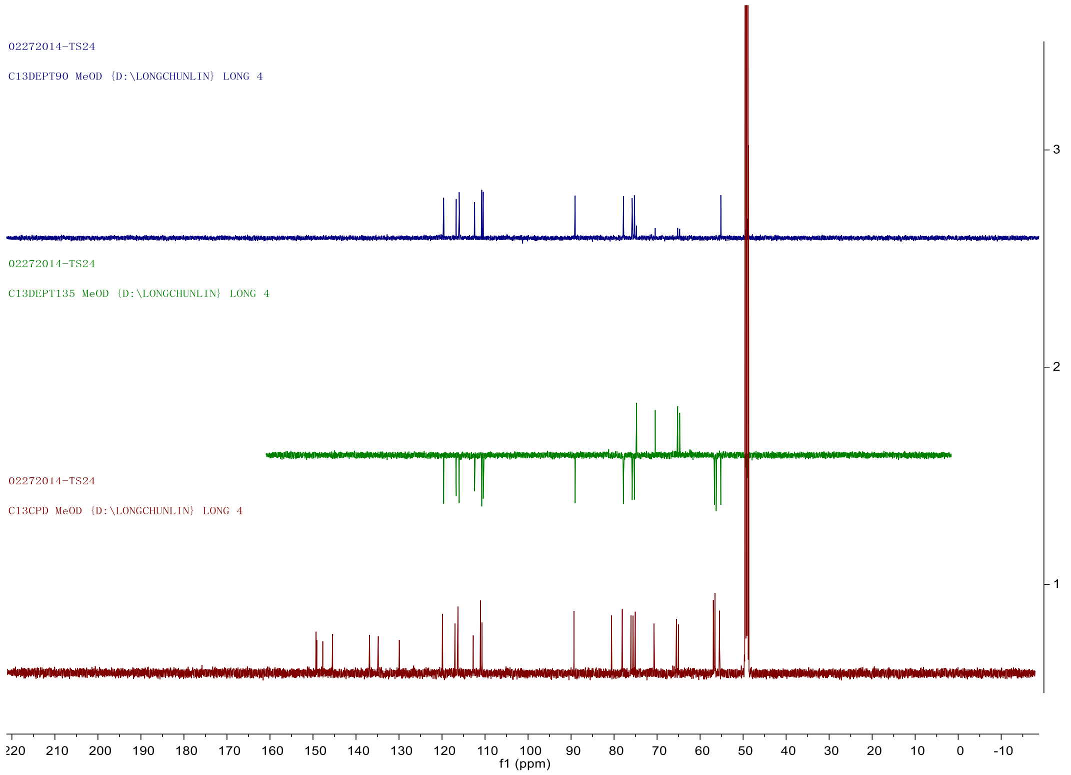

Figure S1-3 ^{1}H-^{1}H-COSY spectrum of compound **1**

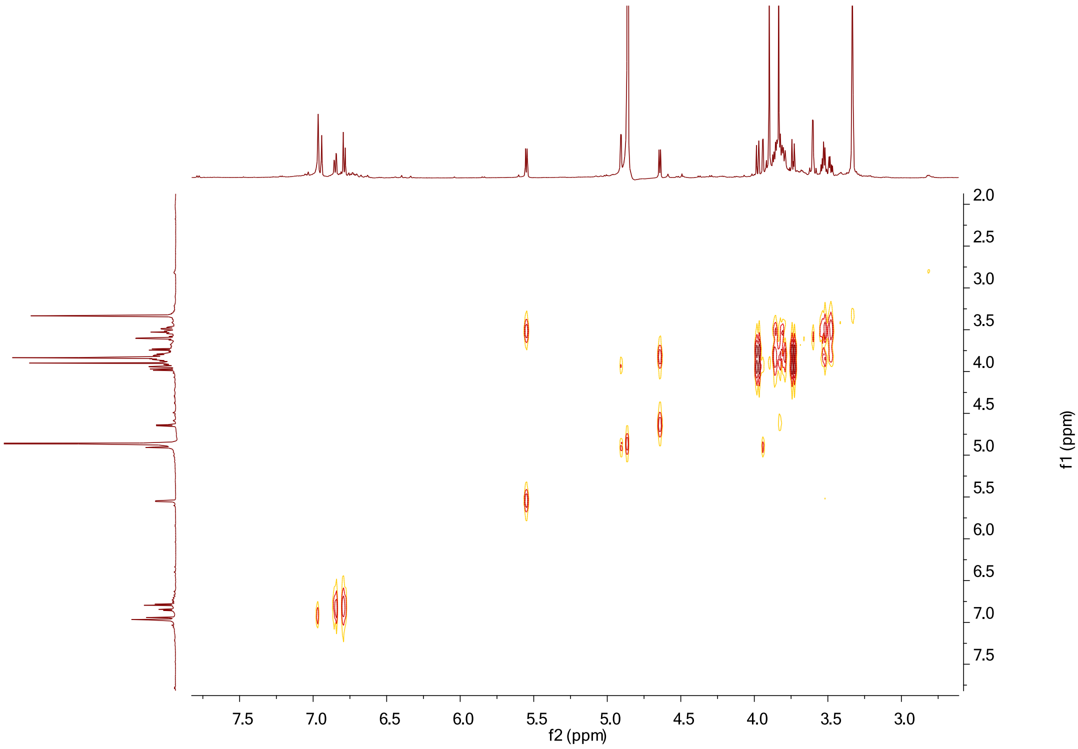

Figure S1-4 HSQC spectrum of compound **1**

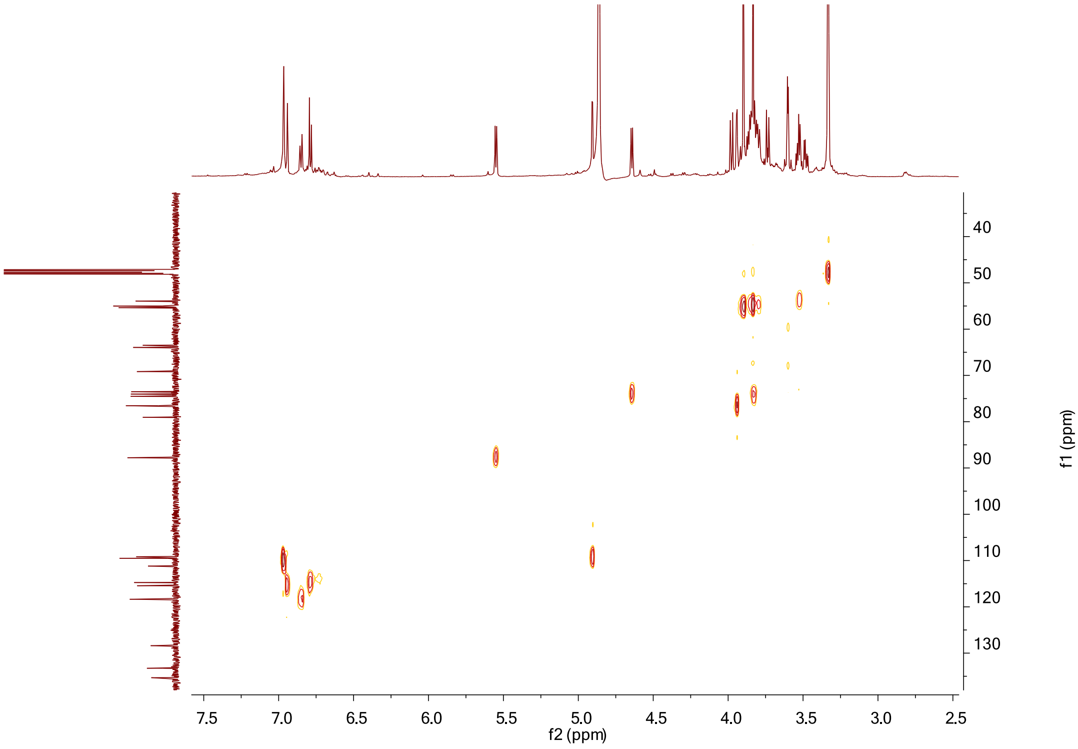

Figure S1-5 HMBC spectrum of compound **1**

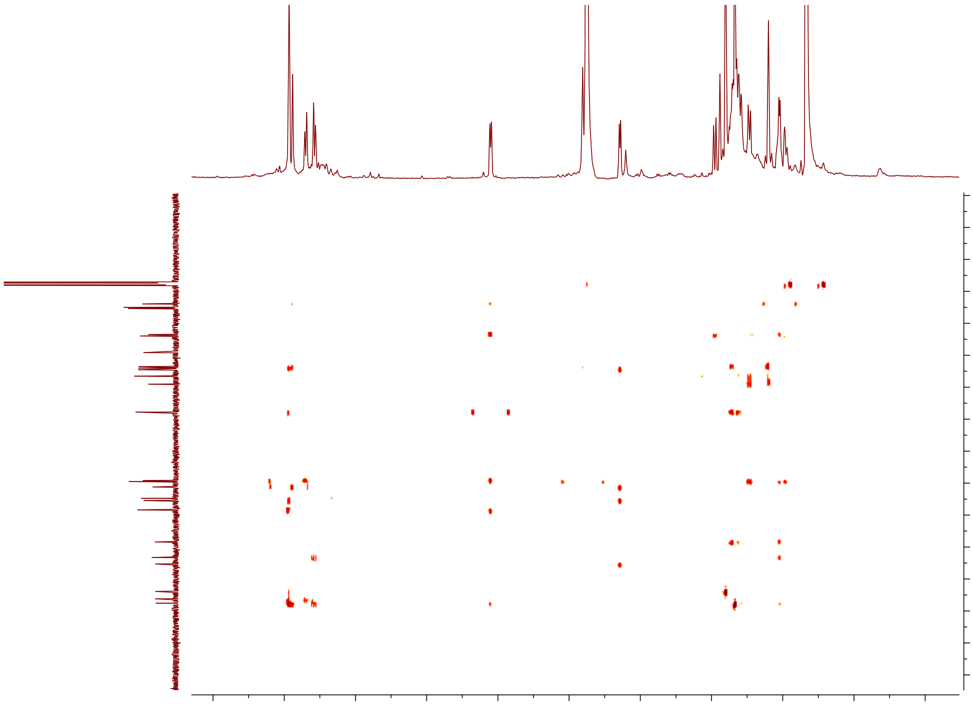

Figure S1-6 NOESY spectrum of compound **1**

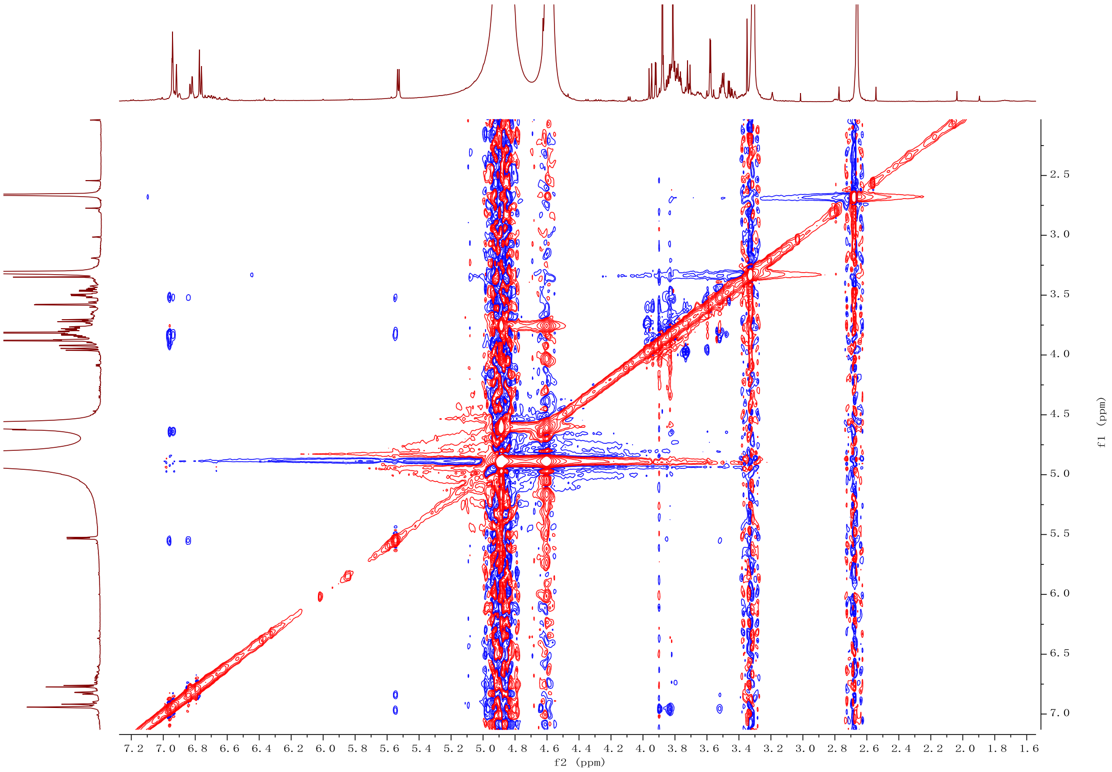

Figure S1-7 HR-LC-TOF-MS positive spectrum of compound **1**

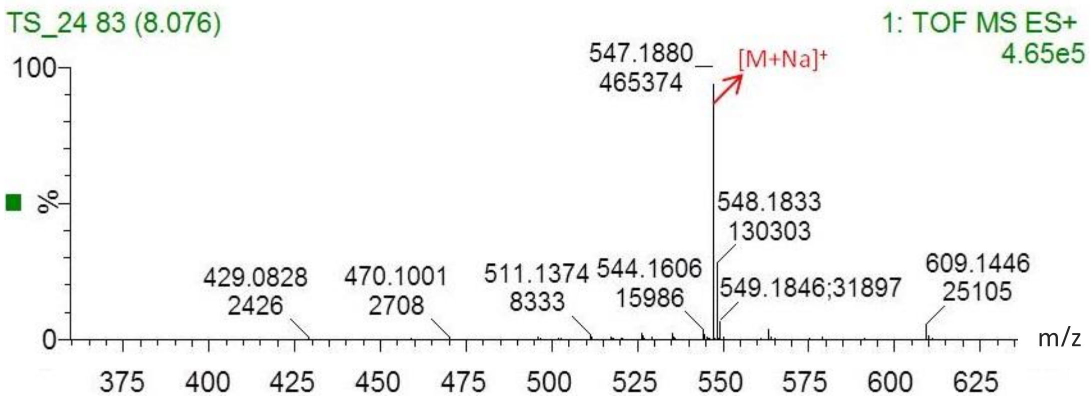

Figure S1-8 IR spectrum of compound **1**

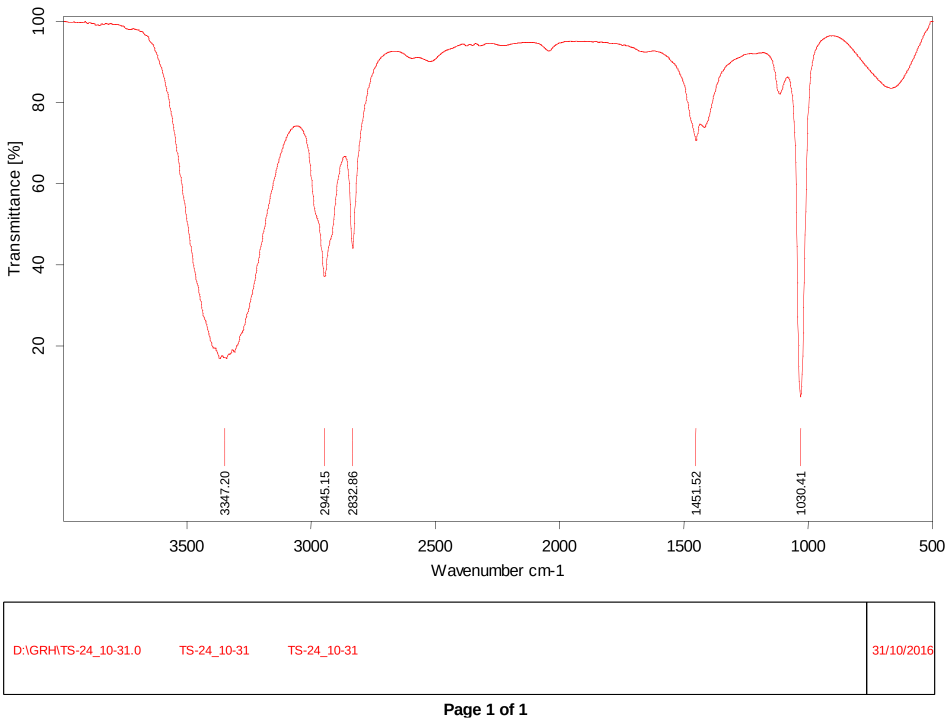

Figure S1-9 UV spectrum of compound **1**

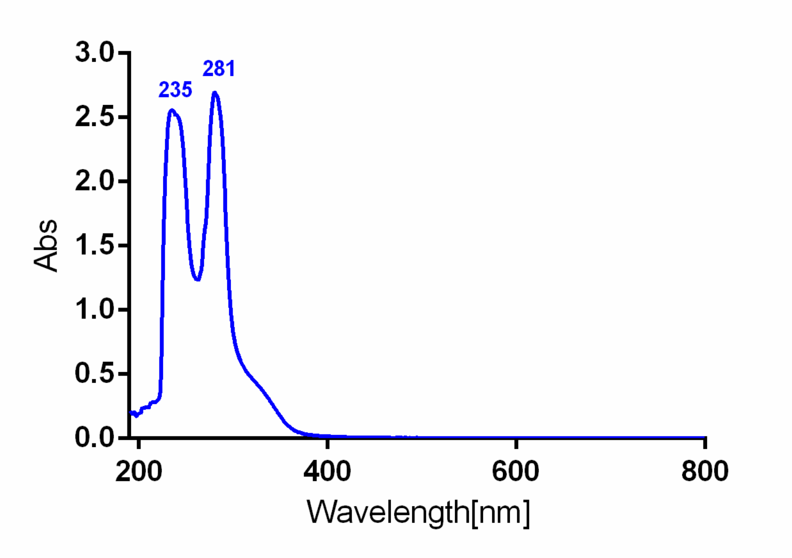

Figure S2. Spectra of compound **2**

Figure S2-1 ^{1}H-NMR spectrum of compound **2**

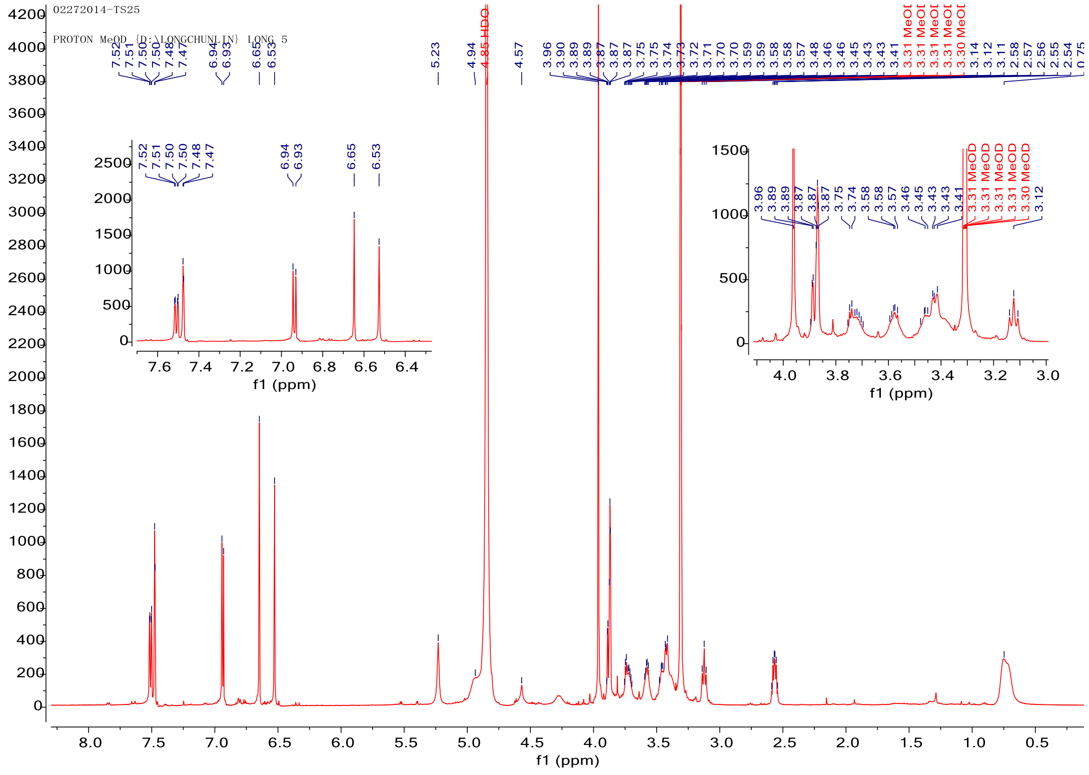

Figure S2-2 ^{13}C-NMR and DEPT spectra of compound **2**

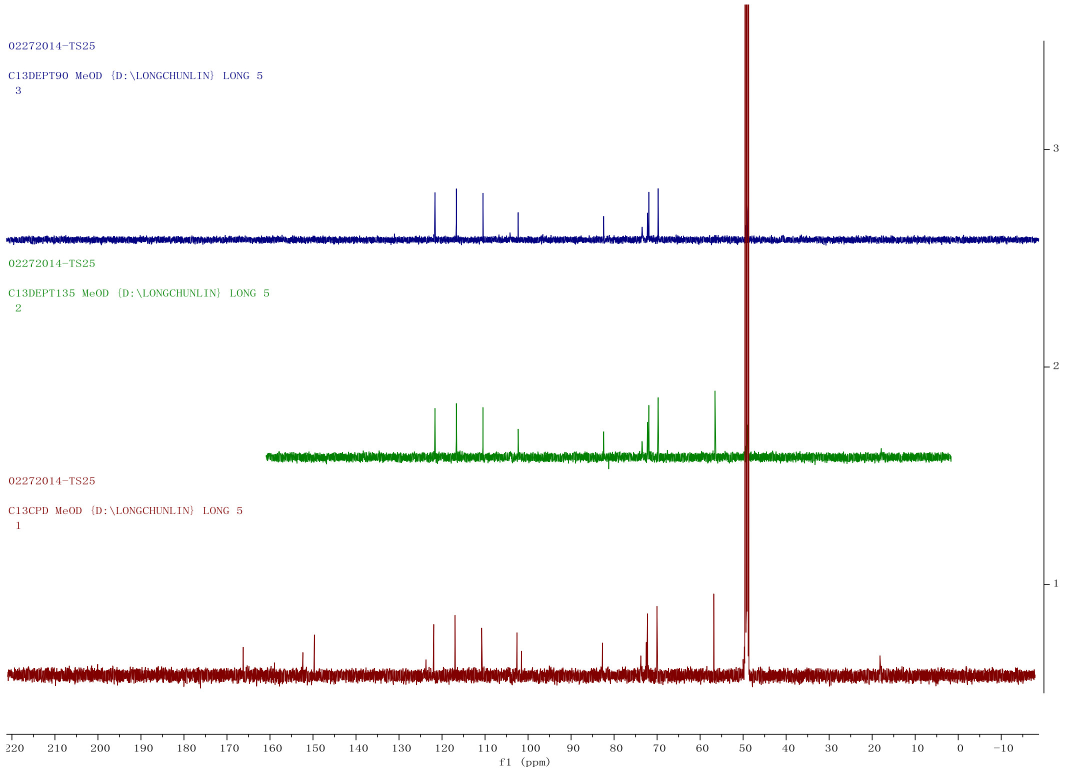

Figure S2-3 HSQC spectrum of compound **2**

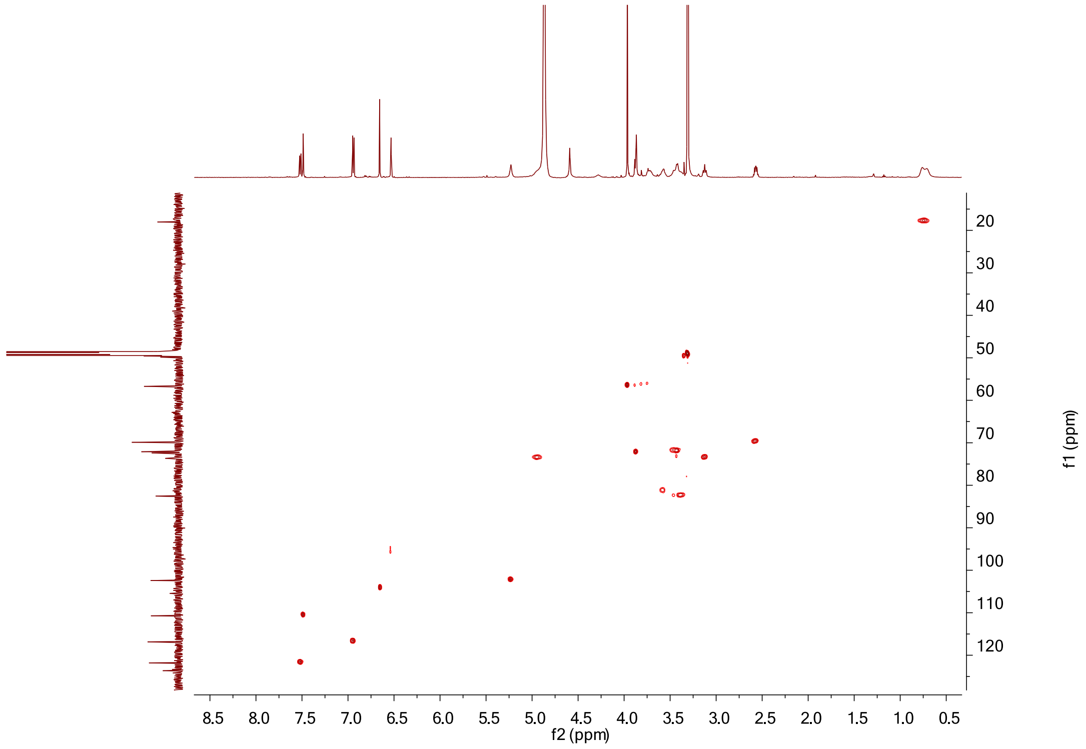

Figure S2-4 HMBC spectrum of compound **2**

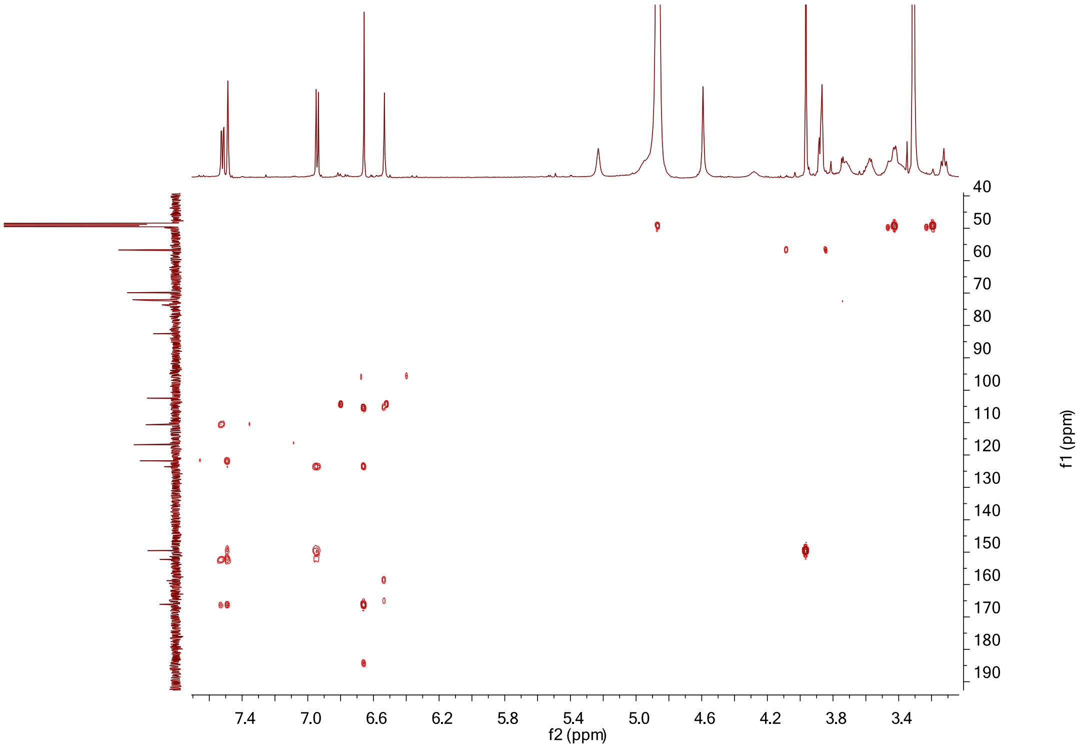

Figure S2-5 NOESY spectrum of compound **2**

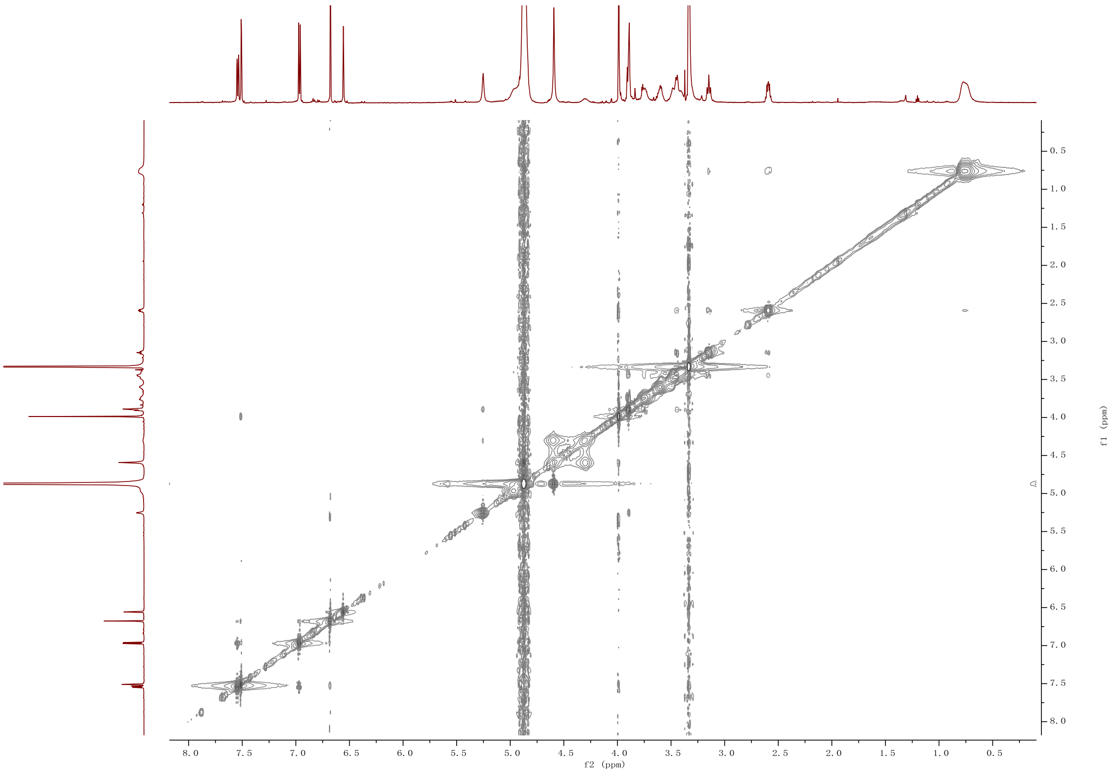

Figure S2-6 HR-LC-TOF-MS positive spectrum of compound **2**

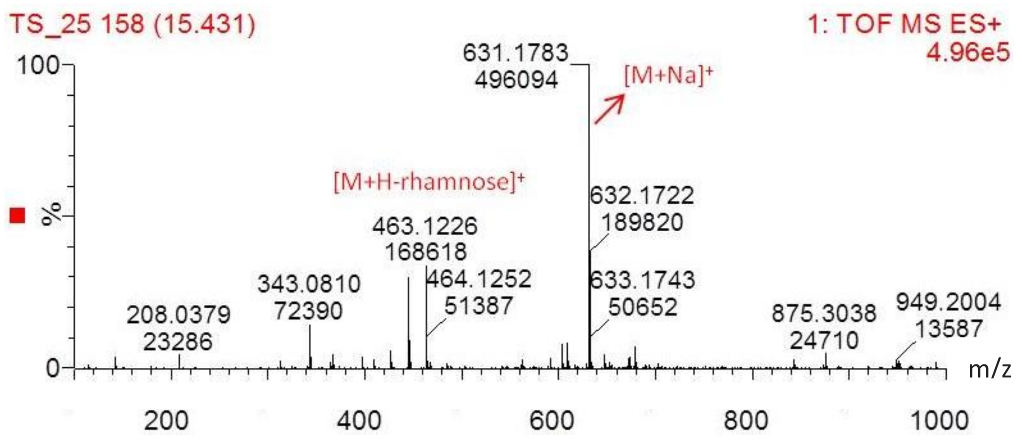

Figure S2-7 IR spectrum of compound **2**

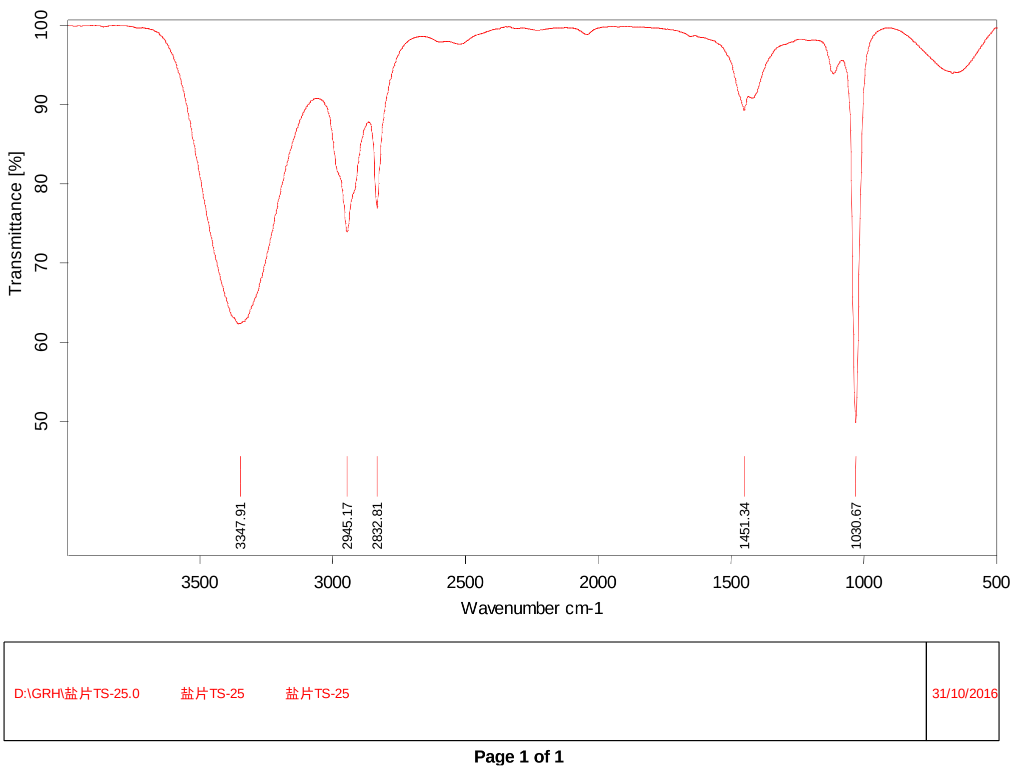

Figure S2-8 UV spectrum of compound **2**

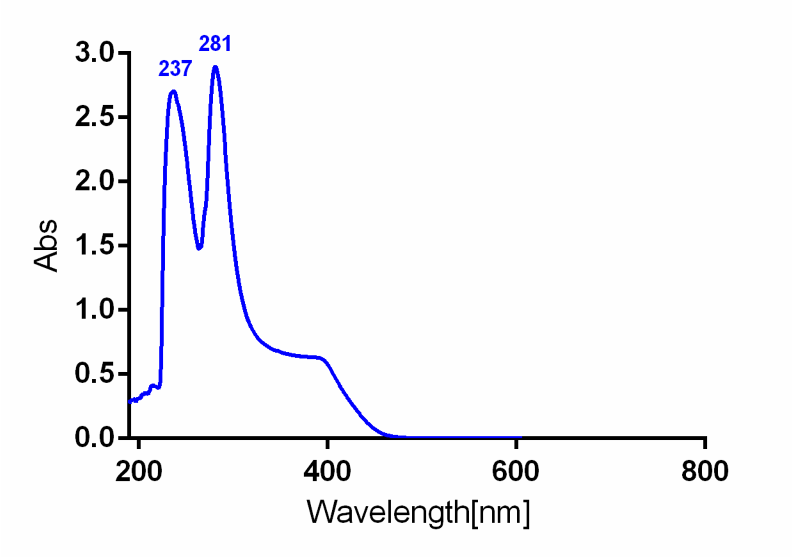

Figure S3. Spectra of compound **3**

Figure S3-1 ^{1}H-NMR spectrum of compound **3**

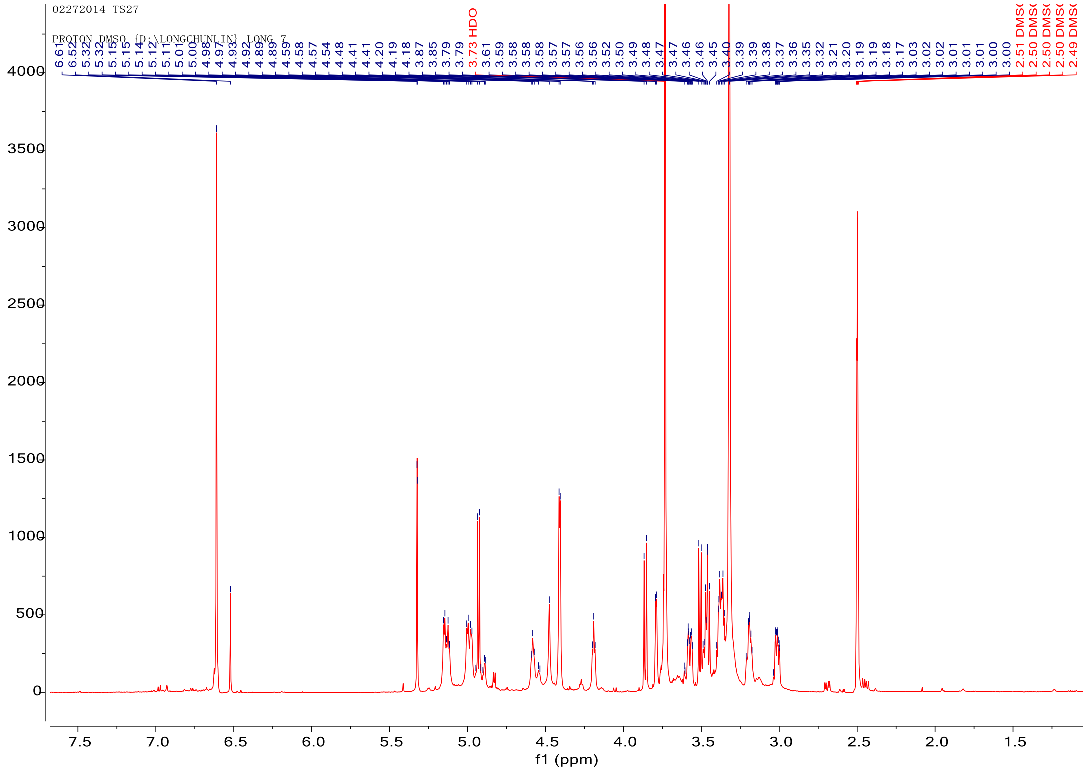

Figure S3-2 ^{13}C-NMR spectrum of compound **3**

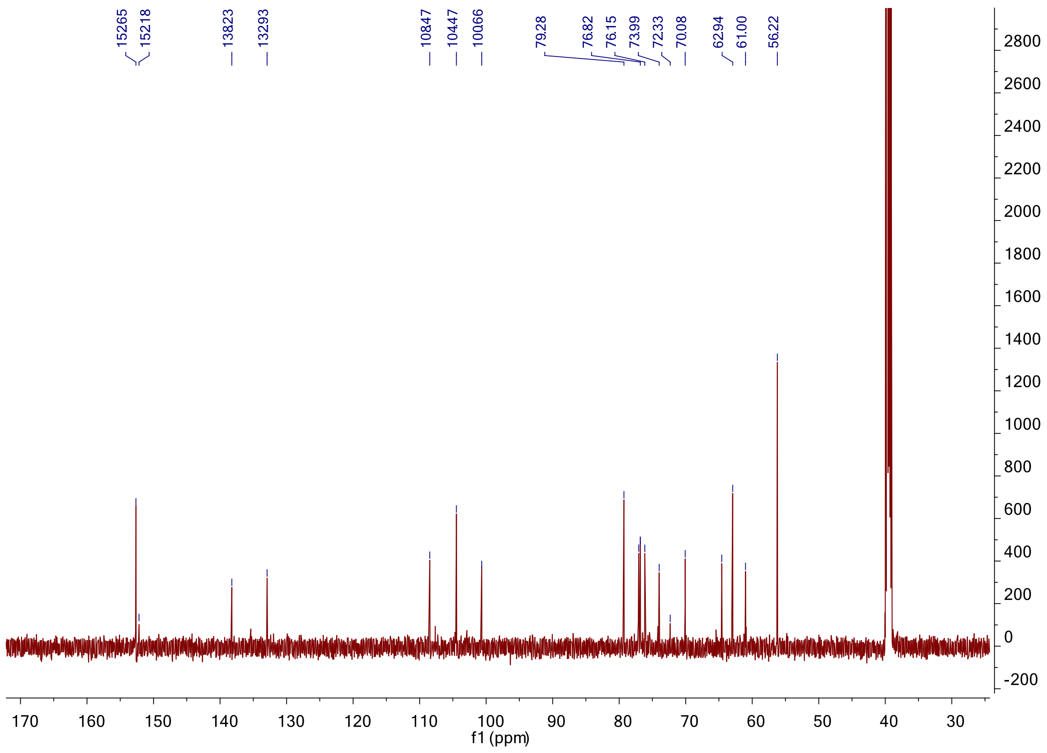

Figure S3-3 HSQC spectrum of compound **3**

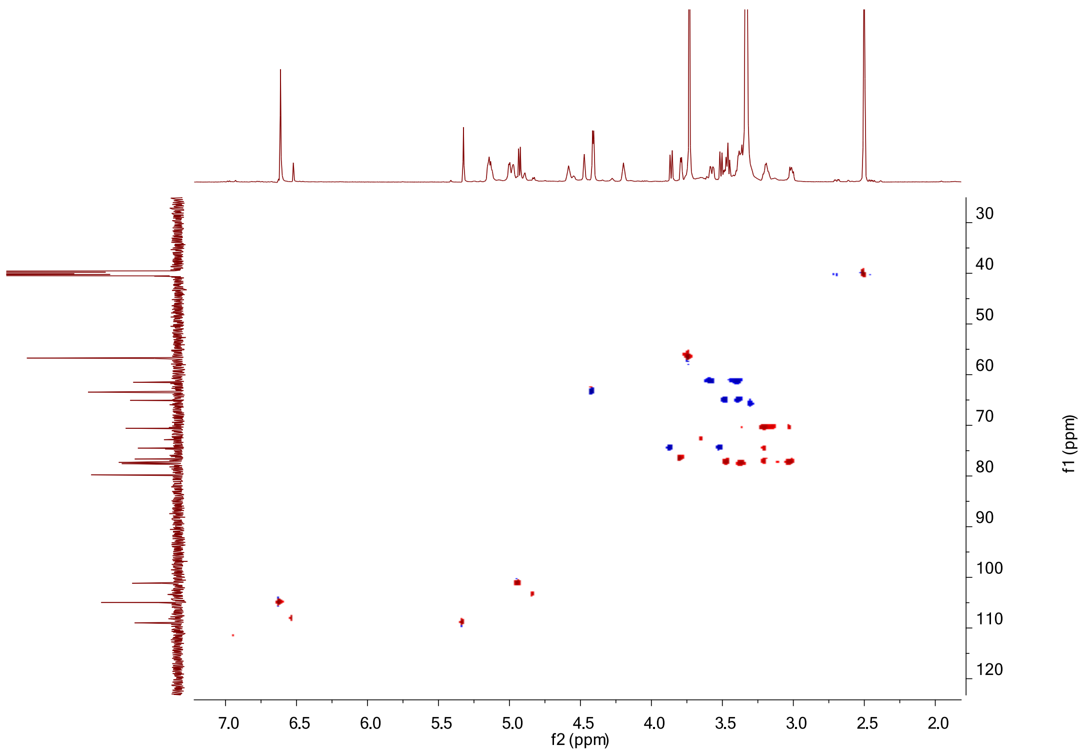

Figure S3-4 HMBC spectrum of compound **3**

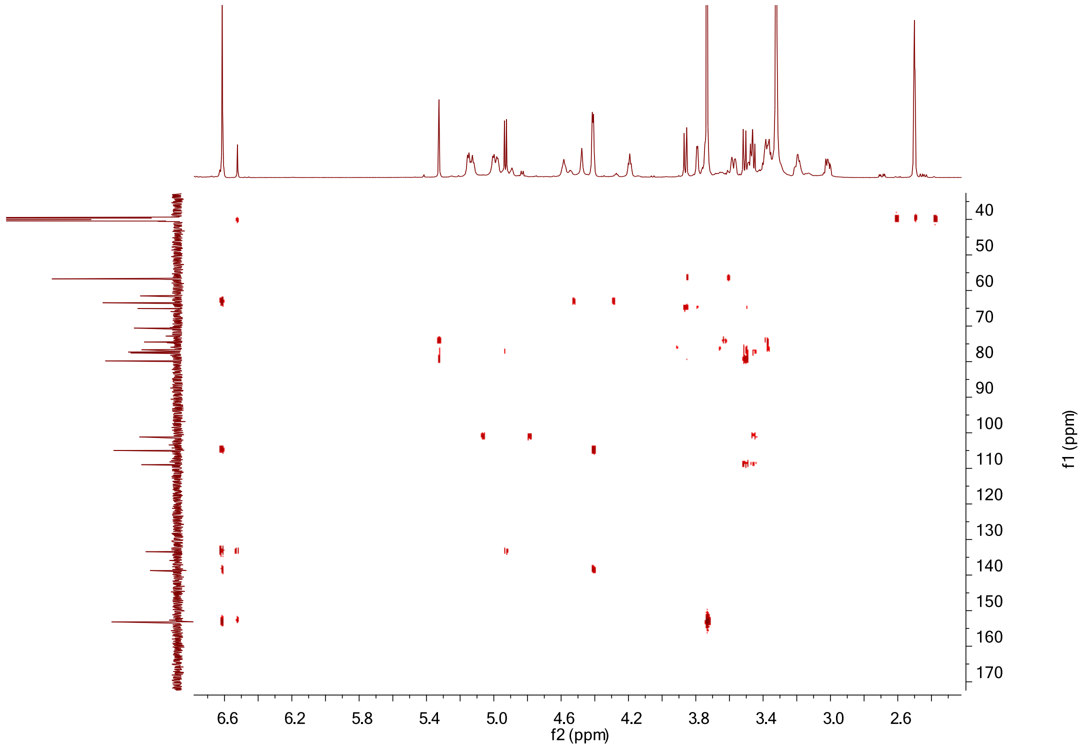

Figure S3-5 ^{1}H-^{1}H COSY spectrum of compound **3**

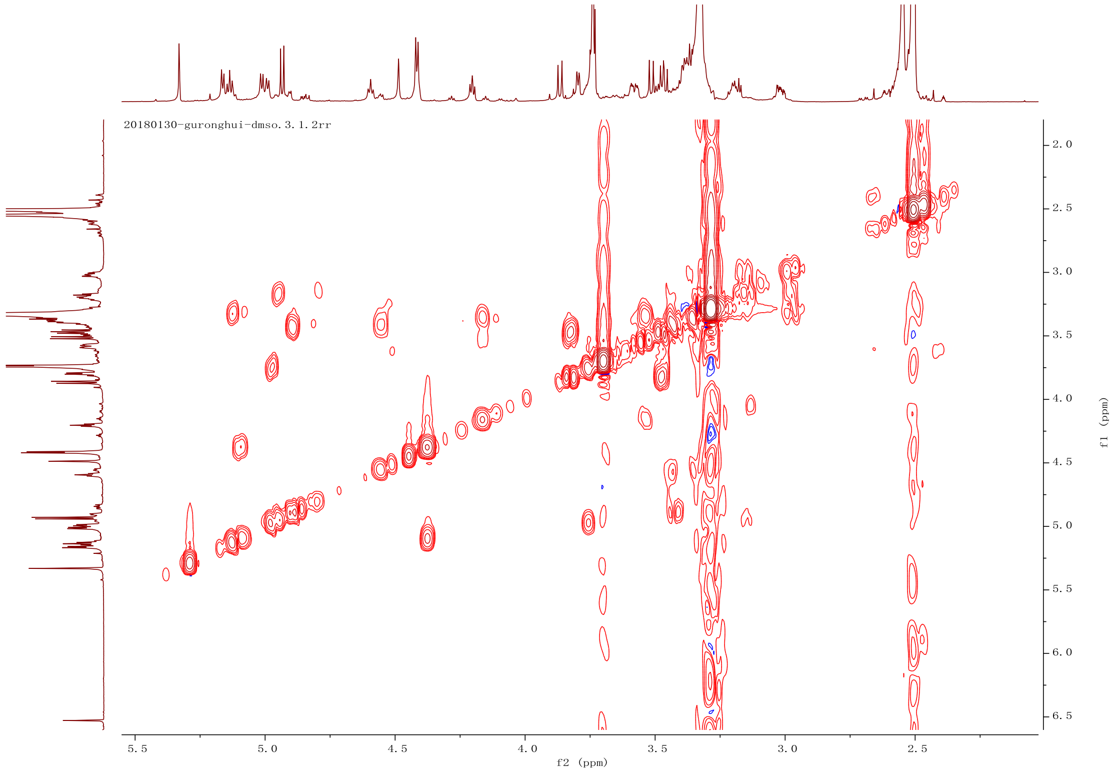

Figure S3-6 HR-LC-TOF-MS positive spectrum of compound **3**

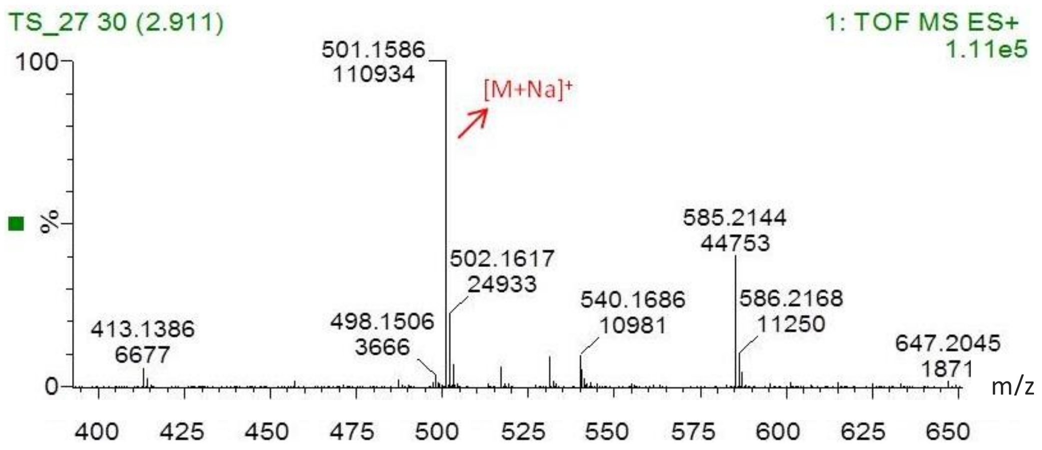

Figure S3-7 IR spectrum of compound **3**

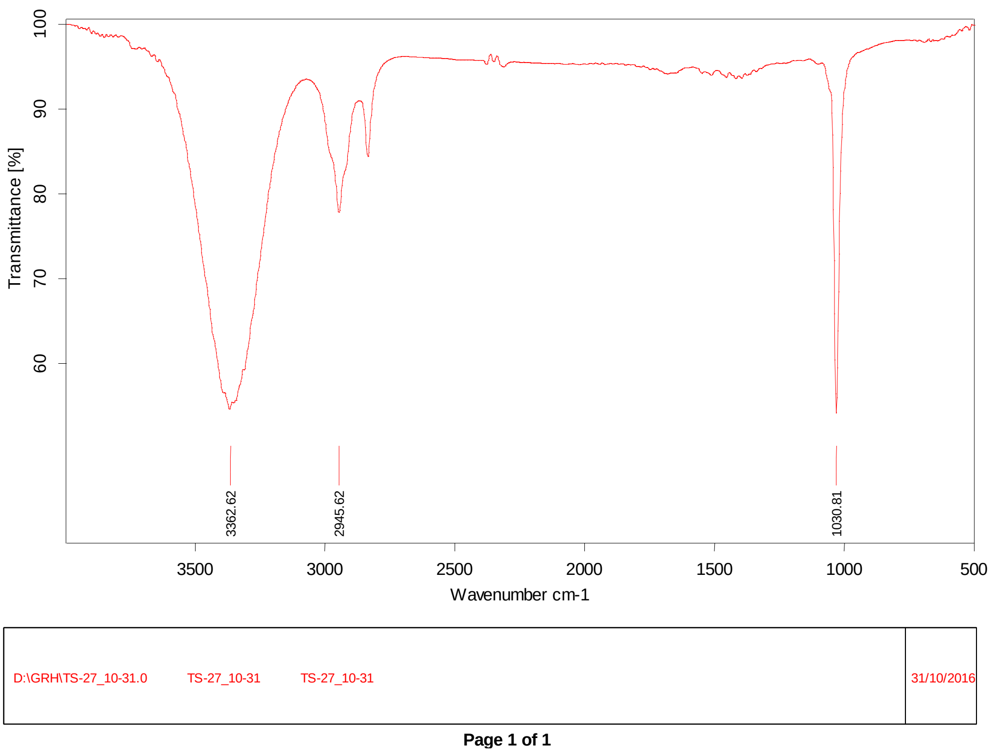

Figure S3-8 UV spectrum of compound **3**

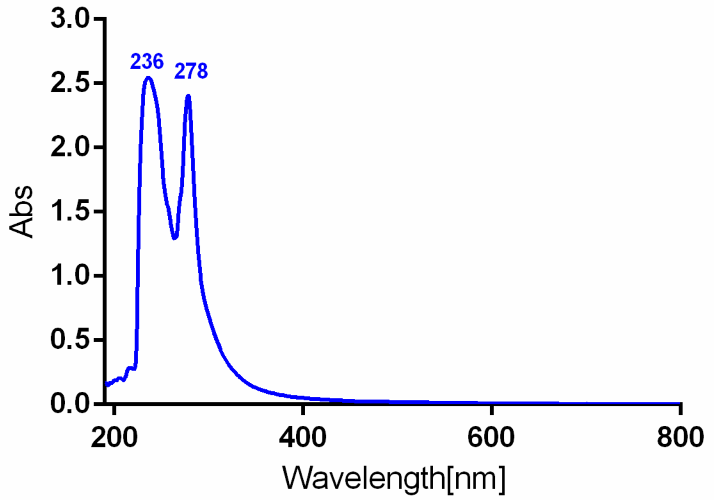

Supplement: Supplementary file 1 — Additional file 1. The details spectra for the identification of compound 1–3. This file includes 1D-NMR, 2D-NMR, HR-MS, IR and UV spectra of compound 1–3. [file 13065_2019_568_MOESM1_ESM.pdf]
